# Transcriptomic profiling of chlorogenic acid and taurine treatment in human skin cells provides insights into cellular senescence mechanisms

**DOI:** 10.3389/fmolb.2026.1748185

**Published:** 2026-03-20

**Authors:** Beomsu Kim, Joong-Gon Shin, In-Shik Hong, Yeeun Ahn, Jung Yeon Seo, Jae Young Shin, Sooyeon Lee, Seung-Hyun Jun, Eui Taek Jeong, Hyeonbin Jo, Mi-So Park, Dan Say Kim, Nae Gyu Kang, Yunkwan Kim, Hong-Hee Won

**Affiliations:** 1 Samsung Advanced Institute for Health Sciences and Technology (SAIHST), Samsung Medical Center, Sungkyunkwan University, Seoul, Republic of Korea; 2 Division of Nephrology, Boston Children’s Hospital, Boston, MA, United States; 3 Kidney Disease Initiative, Broad Institute of MIT and Harvard, Cambridge, MA, United States; 4 R&D Institute, LG Household & Health Care (LG H&H), Seoul, Republic of Korea

**Keywords:** chlorogenic acid, RNA sequencing, skin aging, taurine, transcriptomics

## Abstract

**Background:**

Chlorogenic acid (CGA) and taurine are well-known antioxidant compounds reported to reduce skin cellular senescence. However, the biological mechanisms underlying their skin-protective effects remain unclear.

**Methods:**

In this study, we conducted transcriptome-wide RNA sequencing to profile gene expression changes in human epidermal keratinocytes, melanocytes, and fibroblasts following treatment with CGA, taurine, or their combination. To identify aging-related genes, we integrated evidence from aging databases, perceived-age GWAS, enrichment in aging-related gene ontology and pathways, and drug-gene interaction annotations. Validation of representative genes was performed using quantitative real-time PCR.

**Results:**

A total of 197 differentially expressed genes (DEGs) were identified, of which 62 were prioritized as aging-related DEGs (AR-DEGs) based on their relevance to skin aging anti-senescence-associated pathways, highlighting regulatory transcription factors including *TGFB2*, *ETS1*, and *EGR1*. Co-treatment enhanced the transcriptional effects of CGA and taurine, with several genes exhibiting synergistic responses. Targeted transcriptome-wide association analysis indicated potential links between specific AR-DEGs, such as FST, and phenotypes including perceived age and skin pigmentation.

**Conclusion:**

By identifying key genes and pathways that contribute to cellular longevity in human skin, this study provides molecular insights for developing anti-aging strategies with potential applications in dermatology.

## 1 Introduction

Environmental factors such as ultraviolet (UV) radiation and pollution disrupt cellular homeostasis and accelerate skin aging (Parrado et al., 2019; Krutmann et al., 2021). This leads to various dermatological conditions, including reduced melanocyte viability, impaired keratinocyte differentiation, and increased collagen degradation in fibroblasts, driven by oxidative stress, chronic inflammation, and cellular senescence (Liu et al., 2023). Among these, cellular senescence is known as a central driver of aging (Ho and Dreesen, 2021; Rube et al., 2021; Thau et al., 2025). Senescent cells secrete pro-inflammatory factors and reactive oxygen species (ROS), activating inflammatory cascades and causing oxidative stress accumulation, which accelerate skin aging and reinforce associated pathways (Low et al., 2021; Chin et al., 2023). Given the key role of oxidative stress and inflammation in skin pathophysiology, there is growing interest in bioactive compounds that protect skin cells and mitigate these adverse effects (Michalak et al., 2021; Bjorklund et al., 2022; Kim J. et al., 2024; Lee H. et al., 2024; Lee et al., 2024b; Zhu et al., 2024; Tomas et al., 2025).

Chlorogenic acid (CGA) and taurine, two bioactive compounds with demonstrated antioxidative and anti-inflammatory properties, have been shown to attenuate senescence in skin cells (Qaradakhi et al., 2020; Xue et al., 2022; Yoshimura et al., 2023; Girsang et al., 2024; Liu et al., 2024; Nguyen et al., 2024). Both compounds enhance the activity of endogenous antioxidant enzymes, such as superoxide dismutase and catalase, by modulating the NRF2 and FOXO pathways, reducing ROS levels and mitigating oxidative stress (Sabir et al., 2022; Wang et al., 2022; Milkovic et al., 2023). Additionally, they regulate inflammatory cytokine production by modulating NF-κB and MAPK-mediated signaling pathways (Swiderski et al., 2023; Nguyen et al., 2024). Specifically, CGA has been shown to suppress IL-6, IL-1β, and TNF-α in the NF-κB pathway and increase COL3 expression, protecting fibroblasts from UV-induced damage (Girsang et al., 2021; Huang et al., 2023; Girsang et al., 2024), whereas taurine suppresses IL-1α-induced MMP1 expression in fibroblasts (Yoshimura et al., 2023). These mechanisms suggest that CGA and taurine may significantly contribute to protecting against damaged or senescent skin cells.

Given the shared and distinct mechanisms of CGA and taurine, as well as the potential synergistic effects of combining different antioxidants, co-treatment is anticipated to enhance their individual benefits (Lee H. et al., 2024). Although the individual effects of CGA and taurine on cellular physiology are well documented, their combined effects on skin cells remain largely unexplored. A recent study reported that co-treatment of CGA and taurine suppressed the expression of several inflammatory cytokines (IL-1α, IL-1β, and IL-6) and regulated the expression of genes related to subcutaneous repair and hydration (Lee et al., 2024a), suggesting potential skin-protective effects of the combined treatment. However, previous studies have mainly focused on a limited set of genes without functional annotation or validation using public databases, making it difficult to elucidate the underlying mechanisms of these compounds. To address this gap, a systematic identification of responsive genes and pathways that mediate the anti-senescence effects of CGA and taurine is necessary.

**FIGURE 1:**
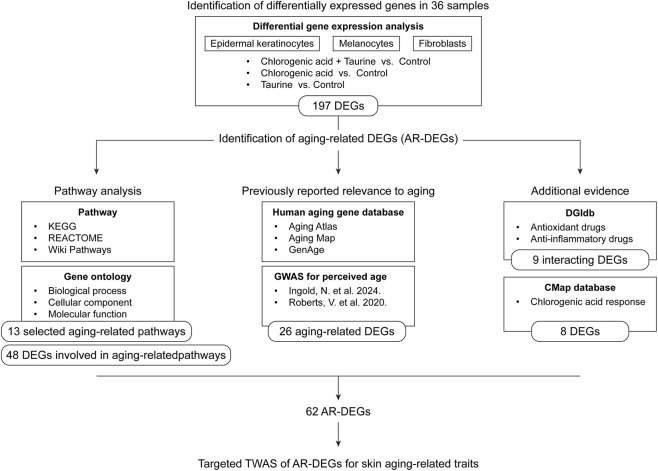
Study workflow diagram. Abbreviations: DEG, differentially expressed gene; AR-DEG, aging-related differentially expressed gene; GWAS, genome-wide association study; DGIdb, Drug–Gene Interaction Database; CMap, Connectivity Map.

The objective of this study was to identify genes that respond to treatment with CGA and taurine, and to elucidate the underlying anti-senescence mechanisms through transcriptomic analysis. To this end, we performed transcriptome-wide RNA sequencing (RNA-seq) to profile gene expression changes in epidermal keratinocytes, melanocytes, and fibroblasts treated with CGA, taurine, or their combination. We identified 197 differentially expressed genes (DEGs), including 62 aging-related DEGs (AR-DEGs) prioritized based on evidence of their relevance to skin aging (Figure 1). These AR-DEGs were associated with various functional categories, including oxygen response, cellular senescence, cell cycle regulation, extracellular matrix organization, and regulation of immune and oxidative stress responses. These findings provide a foundation for the development of anti-aging strategies with potential applications in dermatology.

## 2 Materials and methods

### 2.1 Cell culture and treatment for RNA-seq

Three primary human skin cell lines were used. Normal Human Epidermal Keratinocytes (Cat. no. PCS-200–010; ATCC, Manassas, VA, United States) were cultured in Keratinocyte Growth Medium (Lonza, KGM™ Gold, Cat. no. 00192060; BS, Switzerland) supplemented with 10% fetal bovine serum (Gibco, Waltham, MA, United States), 100 U/mL penicillin, and 100 μg/mL streptomycin (Gibco). Neonatal light-pigmented Human Epidermal Melanocytes (HEMn-LP, Cat. no. C0025C; Thermo Fisher Scientific, Waltham, MA, United States) were cultured in Cascade Biologics™ Medium 254 supplemented with a Human Melanocyte Growth Supplement (Gibco, S0025). Human Dermal Fibroblasts were cultured in Dulbecco’s modified Eagle’s medium (DMEM, Sol Bio Pharm, Gyeonggi-do, Korea) supplemented with 10% fetal bovine serum (Gibco), 100 U/mL penicillin, and 100 μg/mL streptomycin (Gibco). Cells were maintained in a humidified incubator at 37 °C with 5% CO_2_. For the assay, Normal Human Epidermal Keratinocytes and HEMn-LP were seeded at 2 × 10^5^ cells/well, and Human Dermal Fibroblasts at 1 × 10^5^ cells/well in 6-well plates. The cells were maintained for 10 h in a humidified incubator at 37 °C with 5% CO_2_. Subsequently, CGA (Arshine Pharmaceutical Co., Ltd., Changsha, China) and taurine (Qianjiang Yongan Pharmaceutical, Co., Ltd., Qianjiang, China) were administered individually or in combination at appropriate concentrations (CGA, 10 μg/ml; taurine, 1,000 μg/ml; combined treatment, CGA 10 μg/ml + taurine 1,000 μg/ml), followed by incubation for 24 h under the same conditions. The selected concentrations were based on our previous findings (Lee et al., 2024a), which demonstrated that these doses exhibited the most pronounced cumulative effects on skin aging under co-treatment. The treatment time in this study was set to 24 h to ensure comparability with prior literature and to detect the integrated transcriptional effects of each compound (Moghadam et al., 2017; Subramanian et al., 2017; Alves et al., 2019; Lee et al., 2024a). After 24 h, the culture medium was removed and 1 mL of RNAlater (Cat. no. AM7020; Thermo Fisher Scientific) was added to each well to preserve RNA integrity. The plates were then immediately stored at −80 °C in a deep freezer for high-quality RNA preparation.

### 2.2 Bulk RNA-seq profiling

Total RNA was extracted using the TRIzol reagent (Thermo Fisher Scientific), QIAzol® Lysis Reagent (Qiagen, Germany), and RNeasy® Mini Kit (Qiagen), according to the manufacturer’s instructions. The total RNA concentration was measured using the Quant-iT^TM^ RiboGreen RNA Assay (Thermo Fisher Scientific). Total RNA integrity was assessed using a TapeStation RNA ScreenTape (Agilent Technologies, CA, United States). Samples with RNA integrity number >7.0 were used for RNA library construction. A library was independently prepared using 0.5 μg of total RNA for each sample by Illumina TruSeq Stranded Total RNA Library Prep Gold Kit (Illumina, San Diego, CA, United States) following the instructions in the Illumina TruSeq Stranded Total RNA Reference Guide. The libraries were quantified using the KAPA Library Quantification Kit for Illumina Sequencing platforms according to the qPCR Quantification Protocol Guide (KAPA BIOSYSTEMS, MA, United States) and TapeStation D1000 ScreenTape (Agilent Technologies). Total RNA sequencing (RNA-seq) was conducted by Macrogen (Seoul, Korea) using the NovaSeq X platform with 2 × 100 bp paired-end read chemistry (Illumina) (GEO ID: GSE302932).

The nf-core pipeline (v.3.17.0) (Ewels et al., 2020) was used for the alignment, quantification, and quality control of the raw data. RNA-seq reads were aligned to the GRCh38 reference genome obtained from the Broad Institute (Consortium, 2020) using STAR (v.2.7.11b) (Dobin et al., 2013) after filtering out alternate loci (ALT), human leukocyte antigen (HLA), and decoy sequence (Decoy) contigs. Isoform expressions of known Ensembl transcripts were quantified using Salmon (v.1.10.3) (Patro et al., 2017) and GENCODE release 47 (Mudge et al., 2025). QC and generating read counts were performed using the nf-core/rnaseq pipeline with RSeQC, Preseq, Qualimap, dupRadar, DESeq2, Kranken2, and MultiQC (Wang et al., 2012; Daley and Smith, 2014; Love et al., 2014; Ewels et al., 2016; Okonechnikov et al., 2016; Sayols et al., 2016; Wood et al., 2019).

To obtain reliable results, we applied strict QC criteria to the RNA-seq data instead of relying on independent filtering during differential expression tests using DESeq2 (v.1.44.0) (Love et al., 2014). Of the 78,816 generated genes, 60,801 remained after excluding spike-in controls, duplicates, artificial regions, unconfirmed genes, and pseudogenes. For each cell type, genes with read counts less than ten in at least one sample were excluded, resulting in 14,357, 14,445, and 14,528 genes retained in the epidermal keratinocytes, melanocytes, and fibroblasts, respectively. For each treatment group (treated and control) within each cell type, differential expression analysis was performed by comparing each individual sample against the remaining samples within the same group. The Bayesian shrinkage estimator for log_2_ fold change (log_2_FC), derived from the approximate posterior estimation of generalized linear model coefficients of each DEG, was used as the log_2_FC value for all analyses in this study. Genes that passed the Benjamini–Hochberg multiple testing correction (adjusted *P*-value <0.05) and showed differential expression exceeding the suggestive threshold (|log_2_FC| >0.585) within the same group were defined as within-group DEGs. For each cell type, genes identified as within-group DEGs in at least one treatment group were excluded to reduce heterogeneity within the same condition. A total of 14,111, 13,986, and 14,436 genes from epidermal keratinocytes, melanocytes, and fibroblasts, respectively, were included in the subsequent analyses. Principal component analysis (PCA) of the variance-stabilized gene expression data was performed using DESeq2 (v.1.44.0).

### 2.3 Identification of differentially expressed genes

To identify genes responsive to CGA, taurine, and their combined treatment (CGA + Tau), samples treated with each compound were compared to untreated controls using DESeq2 (v.1.44.0) for each skin cell type, without applying independent filtering. Genes that passed the Benjamini–Hochberg multiple testing correction (adjusted *P*-value <0.05) and showed differential expression (|log_2_FC| >1) were defined as DEGs. Log_2_FC values were estimated using the apeglm shrinkage estimation (Zhu et al., 2019).

To test for synergistic effects, we defined two binary indicator variables representing CGA and taurine exposure: C=1 for CGA or CGA + Tau treatment (0 otherwise), and T=1 for taurine or CGA + Tau treatment (0 otherwise). For each gene i and sample j within each cell type, a negative binomial generalized linear model was fitted using DESeq2 (v.1.44.0), $\log\left(q_{ij}\right)=\beta_{i0}+\beta_{iC}C_{j}+\beta_{iT}T_{j}+\beta_{iCT}\left(C_{j}T_{j}\right)$ , where $q_{ij}$ denotes the normalized mean expression. The interaction coefficient $\beta_{iCT}$ corresponds to the deviation of the combined treatment from additivity: $\frac{\beta_{iCT}}{\log2}=\log_{2}{FC}_{i}\left(CGA+Tau\right)-\left[\log_{2}{FC}_{i}\left(CGA\right)+\log_{2}{FC}_{i}\left(Tau\right)\right]$ , where $\frac{\beta_{iCT}}{\log2}$ denotes log_2_FC of the interaction term. For each cell type, we tested the null hypothesis $H_{0}:\beta_{iCT}=0$ using a two-sided Wald test. Genes were considered to exhibit potential synergistic effects of CGA + Tau if the interaction term reached nominal significance (*P*_interaction_ < 0.05) and satisfied a synergy criterion ( $\left|\log_{2}{FC}_{i}\left(CGA+Tau\right)\right|>\left|\log_{2}{FC}_{i}\left(CGA\right)+\log_{2}{FC}_{i}\left(Tau\right)\right|$ , with concordant effect directions). Given the limited statistical power of interaction tests (McClelland and Judd, 1993; Leon and Heo, 2009), genes reaching a less stringent multiple-testing correction threshold (Benjamini–Hochberg adjusted *P*_interaction_ <0.1) were considered to exhibit significant synergistic effects.

### 2.4 Connectivity Map

To provide additional evidence for the identified DEGs, we utilized the Connectivity Map (CMap) database, which offers transcriptomic profiles of human cell lines treated with various perturbations (Subramanian et al., 2017). Using cmapR v.1.16.0 (Enache et al., 2019), we extracted level 5 L1000 signatures, which consist of moderated Z-score vectors (ModZ) as differential gene expression vectors, for each CGA treatment at doses of 10, 3.33, 1.11, 0.37, 0.125, and 0.04 µM in the melanocyte-derived human skin cancer cell line A375. Genes were considered to have CMap supporting evidence if they satisfied either of the following two criteria with consistent directionality in their expression changes: (1) |log_2_FC| >1 and |ModZ| >1.67; or (2) |log_2_FC| >0.585 and |ModZ| >2. Among the identified DEGs, those with significant adjusted *P*-value but modest fold change (0.585 < |log_2_FC| ≤1) were also included if supported by CMap evidence. These genes were included into downstream analyses.

### 2.5 Functional enrichment analysis

Functional enrichment analyses of the identified DEGs with canonical pathways (KEGG, REACTOME, and Wiki pathways) and Gene Ontology (GO) terms (molecular function [GO:MF], cellular component [GO:CC], and biological process [GO:BP]) were performed using gprofiler2 (v.0.2.3) (Kolberg et al., 2020). Pathways and GO terms with gene sets that passed the false discovery rate corrected *P*-value threshold (adjusted *P*-value <0.05) were considered significantly enriched. Among the identified pathways and GO terms, those related to antioxidative, anti-inflammatory, and anti-senescence effects were manually categorized based on functional descriptions and grouped into broader categories according to biological relevance and shared terminology: cellular senescence and oxygen response, cell cycle regulation, extracellular matrix organization, and immune and oxidative stress regulation.

### 2.6 Identification of aging-related DEGs

We defined DEGs as AR-DEGs if they were supported by any of the following evidence of relevance to aging: (1) prior annotation in aging-related databases such as Aging Atlas (Aging Atlas, 2021), Aging Map (Mao et al., 2023), or GenAge (de Magalhaes et al., 2024); (2) proximity (within 500 kb) to genetic variants reaching genome-wide significance (*P*-value <5 × 10^−8^) in previous genome-wide association studies (GWASs) of perceived age (Roberts et al., 2020; Ingold et al., 2024); (3) inclusion in aging-related pathways or GO terms among those significantly enriched in the functional enrichment analysis; or (4) inferred to interact with drugs with antioxidant or anti-inflammatory activity, as annotated in DGIdb v.5.0.4 (Cannon et al., 2024). The regulons within the AR-DEGs were inferred using transcription factor–target interactions with the highest confidence level A from DoRothEA (Garcia-Alonso et al., 2019).

### 2.7 Targeted transcriptome-wide association study of aging-related DEGs

Associations between AR-DEGs and skin aging-related traits, such as perceived age and skin color (CIE LAB values: L*, a*, and b*) were tested using FUSION (released on 2022–02–01) (Taylor et al., 2019), a tool that performs transcriptome-wide association study (TWAS) based on GWAS summary statistics by mapping genes to traits through expression quantitative trait loci (eQTLs). Summary statistics of GWAS for skin color (48,433 individuals of East Asian ancestry) reported by Kim B. et al. (2024) were obtained from the NHGRI-EBI GWAS Catalog (GCST90320257 for L*, GCST90320258 for a*, and GCST90320259 for b*). GWAS summary statistics for perceived age (European ancestry) were obtained from studies by Roberts et al. (2020) (423,992 individuals) and Ingold et al. (2024) (403,945 individuals), available from the University of Bristol data repository (https://data.bris.ac.uk/data/dataset/21crwsnj4xwjm2g4qi8chathha) and Zenodo (https://doi.org/10.5281/zenodo.10554253), respectively. Using cis-eQTLs for AR-DEGs (defined as variants within ±1 Mb of the transcription start site), associations between genes and traits were tested using precomputed gene expression weights from the Genotype-Tissue Expression project (GTEx) v8 (Consortium, 2020) for non-sun-exposed suprapubic and sun-exposed lower leg skin tissues. Genes that passed the Benjamini–Hochberg multiple testing correction (adjusted *P*-value <0.05) for the number of genes in each test group (tissue-trait pair) were considered statistically significant. Among these, gene–trait eQTL mappings were considered reliable when both the Z-score of the top cis-eQTL for the gene and the corresponding GWAS Z-score of that variant were greater than 3.

### 2.8 Quantitative real-time PCR validation of representative AR-DEGs

To quantify the mRNA expression levels, we conducted quantitative real-time PCR (RT-PCR) analysis. Cell culture conditions were the same as those described in Section 2.1. Total RNA was extracted using the AccuPrep® Universal RNA Extraction Kit (Bioneer, Daejeon, Republic of Korea) according to the manufacturer’s instructions. The purity of the extracted RNA (A260/A280) was assessed using the NanoDrop spectrophotometer. Complementary DNA (cDNA) was synthesized by reverse transcription using the AccuPower® RocketScript™ Cycle RT PreMix (Bioneer) on a PCR thermocycler (R&D Systems), according to the manufacturer’s protocol. Quantitative RT-PCR was performed using cDNA obtained from control cells and cells treated with CGA and taurine. The following TaqMan probes were used: *GAPDH* (Assay ID: 4333764F) as an internal control, *TGFB2* (Hs00234244_m1), *ETS1* (Hs00428293_m1), *IL1A* (Hs00899844_m1), and *IL1B* (Hs01555410_m1). The TaqMan™ Universal Master Mix II, with UNG (Applied Biosystems, Waltham, MA, United States) was used for amplification. PCR reactions were performed on the ABI 7500 Real-Time PCR system according to the manufacturer’s protocol. Data were analyzed using ABI software (version 2.3).

### 2.9 Western blot analysis of p16 and p21

The expression levels of p16 and p21 in fibroblasts were evaluated by Western blotting using incubated supernatants and cell lysates. Cells were washed with ice cold PBS and lysed on ice in M-PER buffer (Thermo Fisher Scientific, MA, United States) supplemented with Complete™ protease inhibitor cocktail and phosphatase inhibitor (Roche, Indianapolis, IN, United States). 40 μg of protein was analyzed by Western blotting with appropriate antibodies. Protein bands were detected using a chemiluminescence detector (iB-right FL1500, Thermo Fisher Scientific, MA, United States). Western blotting results were quantified by measuring band intensity using ImageJ software (NIH, MD, United States) for three individual trials. The expression levels of p16 and p21 were normalized to β-actin.

## 3 Results

### 3.1 Transcriptome profiling of skin cell samples

A total of 36 bulk RNA-seq samples were generated from three human skin cell types (i.e., epidermal keratinocytes, melanocytes, and fibroblasts) each treated with one of three compounds (i.e., CGA, taurine, or CGA + Tau), or left untreated as a control. Each treatment condition included three biological replicates per cell type. PCA of overall gene expression profiles showed clear separation among the three skin cell types (Supplementary Figure S1A).

### 3.2 DEGs responsive to CGA and taurine

We independently performed differential gene expression analysis after treatment with each compound in each human skin cell type (Supplementary Figure S2; Supplementary Table S1). In total, 14,111, 13,986, and 14,436 genes from epidermal keratinocytes, melanocytes, and fibroblasts, respectively, were tested to identify DEGs in response to treatment (Supplementary Table S2). Genes with inconsistent expression among samples within the same cell type and treatment conditions (within-group DEGs; see Methods) were excluded prior to analysis, resulting in a more homogeneous transcriptome profile for downstream analysis (Supplementary Figure S1B, C).

**FIGURE 2:**
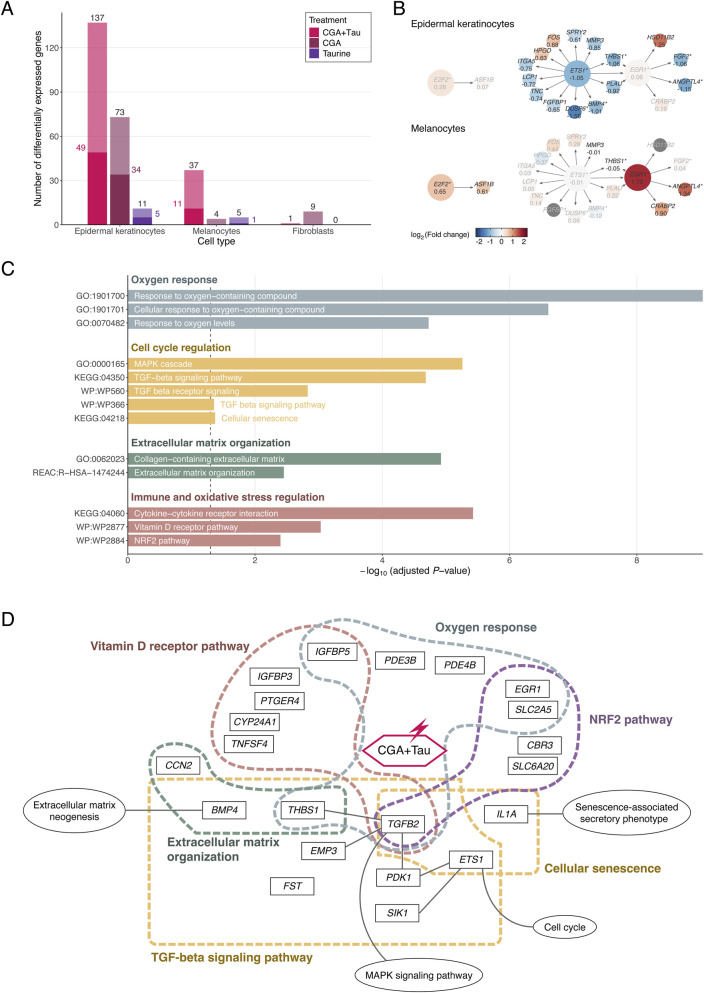
Inferred transcriptomic architecture of antioxidant-responsive DEGs. **(A)**, Number of identified antioxidant-responsive DEGs in each human skin cell type. Bar heights indicate the number of DEGs identified under each treatment condition, with values labeled above each bar. Dark-colored segments represent the subset of AR-DEGs among the antioxidant-responsive DEGs. Bar colors correspond to different treatment conditions. **(B)**, Regulatory network of DEGs inferred using DoRothEA. Circles represent genes with directed edges from transcription factors to their predicted targets based on the DoRothEA regulon. Nodes are colored by log_2_FC from differential expression analysis in epidermal keratinocytes (above) and melanocytes (below). Gene symbols and log_2_FC values are labeled on each node. Genes with |log_2_FC| ≤ 0.585 are shown with lighter colors and gray text. Aging-related DEGs are marked with an asterisk next to the gene names. **(C)**, Antioxidative and anti-inflammatory pathways associated with DEGs. Each bar represents a pathway or GO term (*y*-axis) identified in this study, with the corresponding −log_10_ adjusted *P*-values from gprofiler2 (*x*-axis). The vertical dashed line indicates the significance threshold of adjusted *P*-value = 0.05. **(D)**, A selected molecular model illustrates interaction between treatment of CGA and taurine and genes related with anti-aging mechanisms. Abbreviations: CGA, chlorogenic acid; CGA + Tau, combined treatment of chlorogenic acid and taurine; log_2_FC, Bayesian shrinkage estimator for log_2_ fold change.

We identified 190 DEGs based on an adjusted *P*-value <0.05 and |log_2_FC| >1 (Figure 2A; Supplementary Figure S3A; Supplementary Table S3). In addition, of the 857 genes, seven genes (*E2F2*, *CDC42EP3*, *NRG1*, *CEBPD*, *ABHD4*, *TUBB6*, and *CXCL10*) that met a suggestive threshold (adjusted *P*-value <0.05 and |log_2_FC| >0.585) had supporting evidence from the CMap database (Subramanian et al., 2017) and were also considered DEGs responsive to CGA (Supplementary Figure S4; Supplementary Table S3). Among the 197 DEGs, 147, 41, and 10 were identified in epidermal keratinocytes, melanocytes, and fibroblasts, respectively, with all DEGs being specific to a single cell type, except for *ANGPTL4*. A total of 174, 86, and 16 DEGs were responsive to CGA + Tau, CGA, and taurine, respectively. Of note, five DEGs showed |log_2_FC| greater than 2, including *CCN2* and *KRTAP2-3* (responsive to both CGA + Tau and CGA in epidermal keratinocytes), *PDE3B* and *MARCHF4* (responsive to CGA + Tau in epidermal keratinocytes), and a long non-coding RNA gene, *ENSG00000280800* (responsive to CGA in melanocytes). The majority of DEGs were responsive, either specifically to CGA + Tau (52.7%, 104 DEGs) or to both CGA + Tau and CGA (29.9%, 59 DEGs). Considering the CGA + Tau treatment alone, 88.3% (174 DEGs) of all identified DEGs were detected. In addition, *IL1B* in epidermal keratinocytes and *AK4*, *ANGPTL4*, *BNIP3*, *PKD1*, and *TAC1* exhibited potential synergistic effects of CGA + Tau treatment (*P*_interaction_ <0.05 and satisfying a synergy criterion; see Methods) (Supplementary Table S4; Supplementary Figures S5 and S6). Among these DEGs, *AK4* and *ANGPTL4* passed multiple testing correction under a less stringent threshold (adjusted *P*_interaction_ = 0.051). The DEGs identified in this study showed directional concordance in expression changes across treatments with the three compounds within a given cell type, with CGA + Tau inducing greater fold changes than either compound alone (Supplementary Figure S7). However, when a given compound was used to treat different cell types, distinct sets of DEGs and their highly cell-type-specific fold changes were identified (Supplementary Figure S8).

We identified DEGs encoding transcription factors, such as ETS1, EGR1, and E2F2, and their high-confidence targets using DoRothEA (Garcia-Alonso et al., 2019) (Supplementary Table S5). Among these, *ETS1* and *EGR1* were identified as regulators of other DEGs at the highest confidence level (Figure 2B). *EGR1* was also identified as a high-confidence target of *ETS1*, a DEG responsive in epidermal keratinocytes (CGA + Tau, log_2_FC = −1.05, standard error [SE] = 0.173, adjusted *P*-value = 8.12 × 10^−9^), whereas *EGR1* was regulated specifically in melanocytes (CGA + Tau, log_2_FC = 1.73, SE = 0.407, adjusted *P*-value = 1.25 × 10^−4^; taurine, log_2_FC = 1.52, SE = 0.417, adjusted *P*-value = 5.03 × 10^−3^). Other targets of *ETS1*, including *BMP4*, *DUSP6*, and *THBS1*, showed decreased expression in response to CGA and taurine treatments, which was consistent with the downregulation of *ETS1* in epidermal keratinocytes. Among the targets of *EGR1*, *ANGPTL4* showed increased expression in response to compound treatment in melanocytes (log_2_FC = 1.34, SE = 0.256, adjusted *P*-value = 2.34 × 10^−6^), consistent with upregulation of *EGR1*, but was downregulated in epidermal keratinocytes (log_2_FC = −1.15, SE = 0.174, adjusted *P*-value = 2.52 × 10^−12^). Other *EGR1* targets, including *THBS1* and *FGF2*, showed decreased expression in epidermal keratinocytes.

### 3.3 Functional enrichment analysis of the identified DEGs

To identify the potential mechanisms by which DEGs contribute to antioxidative, anti-inflammatory, and anti-senescence effects, we performed functional enrichment analysis using canonical pathways (KEGG, REACTOME, and WikiPathways) and GO terms. Of the 197 identified DEGs, 126 protein-coding genes were included in the enrichment analysis, which identified 71 canonical pathways and 405 GO terms as significantly enriched gene sets (adjusted *P*-value <0.05) (Supplementary Table S6). Among the enriched pathways and terms of the DEGs, several functional categories relevant to skin aging were identified, such as oxygen response (GO:1901700, GO:1901701, and GO:0070482), cell cycle regulation (cellular senescence [KEGG:04218], MAPK cascade [GO:0000165], and TGF-beta signaling [KEGG:04350, WP:WP560, WP:WP366]), extracellular matrix organization (GO:0062023 and REAC:R-HSA-1474244), and immune and oxidative stress regulation (cytokine-cytokine receptor interaction [KEGG:04060], vitamin D receptor pathway [WP:WP2877], and NRF2 pathway [WP:WP2884]) (Figure 2C). Of note, 48 DEGs were involved in one or more of these skin aging-related functional categories (Supplementary Table S7). Among these, 23 were involved in two or more functional categories. The transcription factors *ETS1* and *EGR1*, along with their targets (*DUSP6*, *BMP4*, *THBS1*, *FGF2*, and *ANGPTL4*), formed a regulon whose components were not part of a single pathway but were instead distributed across multiple pathways related to cellular longevity. *TGFB2* was involved in all major anti-senescence-related functional categories and was intricately interconnected with other DEGs within each category (Figure 2D). *TGFB2* might activate the cell cycle by influencing *PDK1* (GO:0070482 and WP:WP366) and *ETS1* (KEGG:04218 and WP:WP366), which are involved in cellular senescence and oxygen response pathways. Additionally, *TGFB2* is a reported downstream target of *THBS1* (Schultz-Cherry et al., 1994), a DEG involved in the extracellular matrix organization pathway (REAC:R-HSA-1474244 and WP:WP366). *PDE3B* and *CCN2* were both markedly regulated by CGA + Tau treatment in epidermal keratinocytes. *PDE3B* was upregulated (log_2_FC = 2.28, SE = 0.316, adjusted *P*-value = 3.57 × 10^−14^) and involved in the functional category of cellular response to oxygen-containing compound (GO:1901701). *CCN2* was downregulated (log_2_FC = −2.21, SE = 0.122, adjusted *P*-value = 1.92 × 10^−70^) and involved in both the MAPK cascade (GO:0000165) and collagen-containing extracellular matrix (GO:0062023).

### 3.4 Prioritization of AR-DEGs based on supporting evidence

**FIGURE 3:**
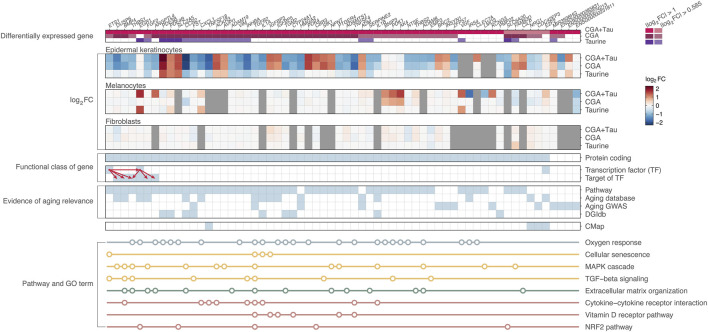
Summary of aging-related aging-related DEGs (AR-DEGs). AR-DEGs are listed along with supporting evidence related to skin aging, including pathway involvement and annotations from public databases and published studies. Arrows in the ‘functional class of gene’ panel indicate the relationship from transcription factors to corresponding target genes. Circles in the ‘Pathway and GO term’ panel represent the involvement of a gene in each gene set. Abbreviations: CGA, chlorogenic acid; CGA + Tau, combined treatment of chlorogenic acid and taurine; log_2_FC, Bayesian shrinkage estimator for log_2_ fold change; TF, transcription factor.

We prioritized 62 AR-DEGs, including 48 involved in skin aging-related functional categories and 14 additional genes, supported by evidence of aging relevance (Figure 3; Supplementary Figure S3B; Supplementary Table S8). For instance, among the DEGs, 16 were previously reported in aging databases such as the Aging Atlas (Aging Atlas, 2021), Aging Map (Mao et al., 2023), and GenAge (de Magalhaes et al., 2024). In addition, 13 were located within 500 kb of previously reported GWAS loci associated with perceived age (Roberts et al., 2020; Ingold et al., 2024). We inferred drugs interacting with antioxidant-responsive DEGs using the DGIdb (Cannon et al., 2024) and identified nine genes, *CCN2*, *CYP24A1*, *FST*, *IL1A*, *IL1B*, *PDE3B*, *PDE4B*, *PMP22*, and *TGM2*, that interact with 18 known antioxidative or anti-inflammatory agents, including apremilast, calcitriol, crisaborole, apremilast, isotretinoin, theophylline, retinoic acid (tretinoin), and vitamins A, D, and E (Supplementary Table S9). A heatmap showing the standardized expression levels of the 62 AR-DEGs across samples within each cell type is provided in Supplementary Figure S9. Among the 62 AR-DEGs, 56 were CGA + Tau-responsive and 52 were differentially expressed in epidermal keratinocytes. Of note, 37.1% (23 of 62 genes) of the AR-DEGs were identified specifically under the CGA + Tau treatment. In addition, five of the six DEGs exhibiting potential synergistic effects of CGA + Tau treatment were prioritized as AR-DEGs, including *AK4* and *ANGPTL*, which passed multiple testing correction in melanocytes (Supplementary Figures S5 and S6). All these synergistic AR-DEGs were involved in oxygen response-related GO terms. Although AR-DEGs identified exclusively under single-treatment conditions were not classified as CGA + Tau-responsive AR-DEGs, they exhibited suggestive expression changes (|log_2_FC| > 0.585) under co-treatment.

### 3.5 Targeted TWAS to explore associations between AR-DEGs and skin aging-related traits

Associations between the predicted expression levels of AR-DEGs and skin aging-related traits (perceived age and skin color [CIE LAB values: L*, a*, and b*]) were tested using a targeted TWAS. A total of 52 AR-DEGs were tested using cis-eQTLs shared between GTEx and the GWAS summary statistics for the skin aging-related traits (Supplementary Table S10). In this targeted TWAS, four AR-DEGs were found to be associated with skin aging-related traits. *BNC2* was associated with perceived age in suprapubic skin tissue (Z-score of TWAS [Z_TWAS_] = −6.20, adjusted *P*-value = 2.91 × 10^−8^) (Supplementary Figure S10). *CDC42EP3* (Z_TWAS_ = 3.45, adjusted *P*-value = 0.010), *NT5E* (Z_TWAS_ = 3.40, adjusted *P*-value = 0.01), and *NPNT* (Z_TWAS_ = −2.83, adjusted *P*-value = 0.042) were associated with perceived age in lower leg skin tissue, but were not considered to have reliable gene–trait associations through eQTLs, as they did not meet the criterion for both the top cis-eQTL Z-score and the corresponding GWAS Z-score (Z-score >3). *ADM* (suprapubic, Z_TWAS_ = −4.77, adjusted *P*-value = 4.70 × 10^−5^; lower leg, Z_TWAS_ = −5.54, adjusted *P*-value = 1.35 × 10^−6^) and *MIR3936HG* (suprapubic, Z_TWAS_ = −3.62, adjusted *P*-value = 4.98 × 10^−3^; lower leg, Z_TWAS_ = −3.01, adjusted *P*-value = 0.029) were associated with perceived age in both skin tissues (Supplementary Figures S11 and S12). *FST*, an AR-DEG downregulated by CGA + Tau treatment, was associated with decreased L* (brightness, Z_TWAS_ = −3.28, adjusted *P*-value = 0.043) and increased a* (redness, Z_TWAS_ = 3.70, adjusted *P*-value = 8.90 × 10^−3^) in lower leg skin tissue (Supplementary Figure S13).

### 3.6 Anti-senescence effect of CGA and taurine

The potential anti-senescence effects of CGA and taurine were additionally validated through *in vitro* assays, including quantitative RT-PCR analysis of representative AR-DEGs for cellular senescence (*TGFB2*, *ETS1*, *IL1A*, and *IL1B*) and Western blot analysis of p16 and p21 proteins. In epidermal keratinocytes, treatment with CGA or CGA + Tau significantly suppressed expression of *TGFB2*, a known pro-senescence factor (CGA, 0.52-fold, *P*-value = 5.96 × 10^−3^; CGA + Tau, 0.48-fold, *P*-value = 5.17 × 10^−3^) (Supplementary Figure S14A). This result was consistent with the transcriptomic profiling, which showed *TGFB2* downregulation under the same conditions. In fibroblasts, changes were observed in other senescence markers, including cell cycle inhibitors and pro-inflammatory cytokines. Specifically, the mRNA level of *IL1A* was decreased following treatment with taurine or CGA + Tau (taurine, 0.68-fold, *P*-value = 0.046; CGA + Tau, 0.59-fold, *P*-value = 0.011) (Supplementary Figure S14B). In parallel, Western blot analysis demonstrated that CGA, taurine, or their combination reduced the protein levels of p16 and p21 in fibroblasts (Supplementary Figure S14C). The effects of CGA and Tau were most pronounced under combined treatment, suggesting a potential enhancement in attenuating cellular senescence at the molecular level. In contrast, *ETS1* expression exhibited changes in the opposite direction at the mRNA level in epidermal keratinocytes (CGA + Tau, 5.29-fold, *P*-value = 0.011) and *IL1B* expression showed modest differences in fibroblasts (CGA + Tau, 0.63-fold, *P*-value = 0.092) following treatment (Supplementary Figures S14A and S14B).

## 4 Discussion

This study aimed to elucidate the molecular mechanisms through which CGA and taurine attenuate senescence in human skin cells. A comprehensive transcriptomic analysis identified 197 DEGs in response to CGA and taurine across epidermal keratinocytes, melanocytes, and fibroblasts, although the seven CMap-supported suggestive candidates require additional validation. Both compounds function through the coordinated regulation of interconnected modes of action linked to cellular longevity mechanisms, particularly regulation of senescence and NRF2 signaling pathways. Notably, we identified three key regulatory factors, *TGFB2*, *EGR1*, and *ETS1*, which may regulate the complex biological network of cellular longevity mechanisms. These results suggested that both compounds act through the coordinated regulation of interconnected transcriptional networks rather than through a single pathway, highlighting the intricate and multifaceted nature of cellular senescence mechanisms.

The effects of CGA and taurine on cellular aging were enhanced under co-treatment. Our transcriptome-wide gene identification results revealed that most AR-DEGs exhibited either unique or stronger responses to the combined treatment. Of note, 37.1% of the AR-DEGs were identified only under co-treatment, supporting enhanced effects of CGA and taurine when used in combination. In addition, five AR-DEGs exhibiting potential synergistic effects of co-treatment, including *AK4*, *ANGPTL4*, *BNIP3*, *IL1B*, and *PKD1*, were consistently involved in oxygen response-related GO terms. These findings may reflect the complementary molecular targets and pathways addressed by both compounds, resulting in more comprehensive protection against damaged or senescent skin cells than either compound alone. The changes in DEGs expression were consistent within a given cell type but varied across cell types, suggesting cell type-specific regulatory dynamics. *ANGPTL4* was the only DEG that responded to CGA and taurine treatment in more than 1 cell type, showing opposite directions of expression change—downregulation in epidermal keratinocytes and upregulation in melanocytes. Given that *ANGPTL4* is a known target of *EGR1* (Yang et al., 2024), an AR-DEG specifically upregulated in melanocytes, this bidirectional response may reflect cell type-specific regulatory dynamics. These findings suggest shared mechanisms underlying the cumulative effects of CGA and taurine, which appear to be cell type-dependent and mediated by multilayered regulatory pathways rather than direct transcriptional responses.

The cell type-specific regulatory effects of CGA and taurine were also observed through the modulation of transcription factor target regulons. Specifically, the transcription factors *ETS1* and *EGR1* have distinct roles in different skin cell types: *ETS1* inhibits terminal differentiation and induces matrix metalloproteinase and innate immune mediators in keratinocytes (Nagarajan et al., 2010), while *EGR1* regulates a-MSH-mediated tyrosinase gene transcription in melanocytes (Shin et al., 2019). Genes targeted by *ETS1* and *EGR1* in the skin, including *DUSP6*, *BMP4*, *THBS1*, *FGF2*, and *ANGPTL4*, showed varied responses to CGA and taurine treatment across skin cell types. Importantly, the genes constituting these regulons influenced various pathways related to cellular longevity, such as cellular senescence and extracellular matrix organization, rather than clustering in a single shared pathway. These results demonstrated that *ETS1* and *EGR1* mediate multiple functional mechanisms affecting cellular aging through complex and multilayered biological pathways in different cell types.

*TGFB2*, a DEG involved in multiple biological pathways associated with cellular aging, is emerging as an important regulator due to its broad influence on cell proliferation, immune modulation, and extracellular matrix dynamics (Massague, 2012). In aging skin, *TGFB2* expression is markedly dysregulated in dermal tissues, contributing to decreased tissue regeneration capacity and altered inflammatory responses (Zhang et al., 2019). *TGFB2* exhibits its functional impact through interaction with several genes. *THBS1* was a DEG involved in the TGF-beta signaling pathway, oxygen response-related GO terms, and extracellular matrix organization. THBS1 promotes collagen assembly and tissue remodeling through its binding to collagen (Tan and Lawler, 2009; Murphy-Ullrich, 2019), and is also involved in O-glycosylation of proteins containing thrombospondin type 1 repeat domains (REAC:R-HSA-5173214) (Supplementary Table S6). Modulation of these pathways may exert anti-glycation effects by stabilizing extracellular matrix protein networks and preventing abnormal collagen cross-linking (Sajithlal et al., 1998). *FST* was another DEG involved in the TGF-beta signaling pathway. The downregulation of *FST* after CGA and taurine treatment provides additional mechanistic insights. Notably, increased expression of *FST* was associated with decreased L* (brightness) and increased a* (redness) in our targeted TWAS, which further supports the involvement of *FST* in the facial skin aging process. Although *BNC2*, *ADM*, and *MIR3936HG* are not involved in established aging pathways, their potential associations with perceived age were identified. These findings suggested that treatment with CGA and taurine influences the phenotypic features of skin aging, and that our transcriptomic approach may capture implicit aging-related mechanisms.

The key regulatory factors *TGFB2*, *ETS1*, and *EGR1* identified in this study provide mechanistic insight into how CGA and taurine may jointly modulate senescence-related processes in human skin cells. *TGFB2*, a key mediator of TGF-beta signaling, has been implicated in the attenuation of TGF-beta-mediated senescence-associated secretory phenotype (SASP) activity and extracellular matrix remodeling across multiple biological contexts when downregulated (Tominaga and Suzuki, 2019; Bhardwaj et al., 2025). *ETS1*, which was involved in the TGF-beta signaling pathway in our study, has been associated with differentiation, proliferation, oxidative stress, and cellular senescence (Xiao et al., 2022; Geng et al., 2025). In the epidermal context, *ETS1*-mediated inhibition of keratinocyte terminal differentiation may compromise skin barrier integrity, as this process is essential for formation of the cornified layer and maintenance of keratinocyte homeostasis (Nagarajan et al., 2010). *EGR1*, a target of *ETS1*, has been reported as an oxidative stress-responsive transcription factor (Shin et al., 2017). In this study, genes targeted by *EGR1* were enriched in pathways related to the MAPK cascade and extracellular matrix organization. Given the central role of MAPK signaling in transducing oxidative and inflammatory cues (Kyriakis and Avruch, 2012), *EGR1* may act as a transcriptional mediator linking upstream stress signals to downstream extracellular matrix remodeling processes. Overall, the regulatory factors identified in this study indicate that transcriptional responses to CGA and taurine converge on coordinated modulation of stress-responsive programs relevant to skin aging.

The potential interactions of nine AR-DEGs, including *FST*, *PDE3B*, and *CCN2*, with known antioxidant or anti-inflammatory agents were identified in the DGIdb database. Retinoic acid interacts with *FST*; pentoxifylline and theophylline interact with *PDE3B*; and curcumin interacts with *CCN2*. Retinoic acid is recognized for its antioxidative and tissue-remodeling properties as well as its ability to enhance skin cell turnover and radiance (di Masi et al., 2015; Bohm et al., 2025). Pentoxifylline influences the production of pro-inflammatory cytokines in keratinocytes (Bruynzeel et al., 1998). Theophylline exerts skin-protective effects by enhancing antioxidant defenses, preserving the extracellular matrix, and increasing melatonin production and stem cell marker expression (Bertolini et al., 2020). Curcumin exhibits therapeutic potential in inflammatory skin conditions such as psoriasis, acne, infections, and dyspigmentation (Nguyen and Friedman, 2013; Kasprzak-Drozd et al., 2024). These findings suggest that compounds that act through common molecular mechanisms shared between CGA and taurine have antioxidant and anti-inflammatory effects. Therefore, further research is required to reposition or screen additional compounds to improve cellular longevity.

The observed decreases in p16 and p21 protein levels, along with the modulation of AR-DEG expression, suggest that CGA and taurine may influence early molecular events in the senescence program prior to phenotypic changes become evident (Kumari and Jat, 2021; Wagner and Wagner, 2022). These observations underscore the complexity of skin aging mechanisms and highlight the need for integrative, multi-layered analytical approaches, such as those employed in this study, to more comprehensively characterize cellular senescence. However, the absence of phenotypic validation of the anti-senescence effects, such as SA-β-gal staining, remains a limitation of this study. In addition, limited replication of RNA-seq findings by quantitative RT-PCR may reflect the reduced sensitivity of transcriptomic profiling for detecting low-abundance transcripts. Further validation is required to clarify whether each ingredient exerts anti-senescence effects and lead to phenotypic improvement in cellular senescence, depending on treatment concentration or duration.

This study has several limitations. First, although transcriptome-wide gene expression changes induced by CGA and taurine were profiled across three primary human skin cell types, the findings were derived from *in vitro* conditions with a limited sample size and a single time point, which may not fully recapitulate the complexity of *in vivo* skin tissue and may miss earlier or delayed transcriptional responses. Second, rigorous validation of synergistic effects under co-treatment will necessitate larger-scale studies, as interaction tests require considerably larger sample sizes than main effect models to achieve comparable statistical power (McClelland and Judd, 1993; Leon and Heo, 2009). Third, while bulk RNA sequencing per cell type may capture cell-type-specific molecular characteristics, single-cell analysis in future studies may have a higher resolution at the cell subpopulation level. Finally, the interpretation of gene–trait associations was based on publicly available GWAS and eQTL data, which were predominantly derived from populations of European ancestry. This population bias may limit the generalizability of our findings, particularly in non-European contexts. Conducting a large-scale GWAS of skin aging-related traits in diverse ancestries could facilitate the discovery of gene–trait associations relevant to the cellular longevity mechanisms of action of CGA and taurine.

Despite these limitations, our findings provide a transcriptomic characterization of CGA and taurine responses in three primary human skin cell types. The identification of key gene expression changes induced by CGA, taurine, and their combination highlights both enhanced effects under combined treatment and cell type-specific responses. Through integrative analyses incorporating pathway enrichment analyses, external databases, and validation in *in vitro* assays, we prioritized genes and pathways with potential relevance to skin aging.

In conclusion, our findings contribute to a better understanding of the molecular mechanisms underlying cellular responses to CGA and taurine in the skin and may further inform the development of targeted strategies for dermatological interventions and cellular longevity.

## Data Availability

The summary statistics of GWAS for perceived age in UK Biobank European participants can be downloaded at https://doi.org/10.5523/bris.21crwsnj4xwjm2g4qi8chathha (Roberts et al., 2020) and https://zenodo.org/records/10554253 (Ingold et al., 2024). The summary statistics of GWAS for skin color in East Asian populations can be downloaded at the NHGRI-EBI GWAS Catalog (GCST90320257; https://www.ebi.ac.uk/gwas/studies/GCST90320257, GCST90320258; https://www.ebi.ac.uk/gwas/studies/GCST90320258, and GCST90320259; https://www.ebi.ac.uk/gwas/studies/GCST90320259) (Kim B. et al., 2024). The Connectivity Map (CMap) resources can be downloaded at https://clue.io. The accession numbers for the processed RNA-seq data from the skin samples used in this study are available at the National Center for Biotechnology Information/Gene Expression Omnibus under repository accession number Gene Expression Omnibus (GSE302932; https://www.ncbi.nlm.nih.gov/geo/query/acc.cgi?acc=GSE302932). United Kingdom Biobank data were obtained under application no.33002.
