# Supplementary material for: Transcriptomic profiling of chlorogenic acid and taurine treatment in human skin cells provides insights into cellular senescence mechanisms
Source: Front Mol Biosci. 2026 Mar 20;13:1748185. doi: 10.3389/fmolb.2026.1748185 (PMC13047320; doi:10.3389/fmolb.2026.1748185)
Supplement: Supplementary file 1 [file Supplementaryfile1.pdf]

Supplementary Material

Supplemental Information

Supplemental Figures

Supplemental Fig. S1. Principal component analysis (PCA) of gene expression.

Supplemental Fig. S2. Volcano plots of differential gene expression for each treatment in each human skin cell type.

Supplemental Fig. S3. Number of differentially expressed genes (DEGs) identified in this study.

Supplemental Fig. S4. DEGs having supporting evidence from the Connectivity Map (CMap).

Supplemental Fig. S5. Synergistic effects across the identified DEGs.

Supplemental Fig. S6. DEGs exhibiting potential synergistic effects.

Supplemental Fig. S7. Comparison of $\log_2$ fold changes in the identified DEGs between treatments within each cell type.

Supplemental Fig. S8. Comparison of $\log_2$ fold changes in the identified DEGs between cell types within each treatment.

Supplemental Fig. S9. Heatmap showing the expression levels of the 62 aging-related DEGs (AR-DEGs).

Supplemental Fig. S10. Regional plots of the GWAS for perceived age and eQTLs for BNC2.

Supplemental Fig. S11. Regional plots of the GWAS for perceived age and eQTLs for ADM.

Supplemental Fig. S12. Regional plots of the GWAS for perceived age and eQTLs for MIR3936HG.

Supplemental Fig. S13. Regional plots of the GWAS for perceived age and eQTLs for FST.

Supplemental Fig. S14. Anti-senescence effects of CGA and taurine and validation of representative AR-DEGs.

Supplemental Tables

Supplemental Table S1. Candidate genes identified using DESeq2 (adjusted *P*-value < 0.05).

Supplemental Table S2. Number of genes tested for differential expression in each cell type.

Supplemental Table S3. Differentially expressed genes (adjusted P-value < 0.05 and $|\log_2$ fold change$| > 1$, or $|\log_2$ fold change$| > 0.585$ with supporting evidence from the CMap database).

Supplemental Table S4. Synergistic effect testing of differentially expressed genes.

Supplemental Table S5. Transcription factors encoded by identified DEGs and their high-confidence targets identified from DoRothEA.

Supplemental Table S6. Enriched pathways and GO terms identified using gprofiler2.

Supplemental Table S7. Antioxidative and anti-inflammatory pathways associated with identified DEGs.

Supplemental Table S8. A total of 62 aging-related DEGs (AR-DEGs) with annotations.

Supplemental Table S9. Potential interactions of DEGs with known antioxidant or anti-inflammatory agents from the DGIdb.

Supplemental Table S10. Associations between AR-DEGs and skin aging-related traits tested using FUSION.

1 Supplementary Figures and Tables

1.1 Supplementary Figures

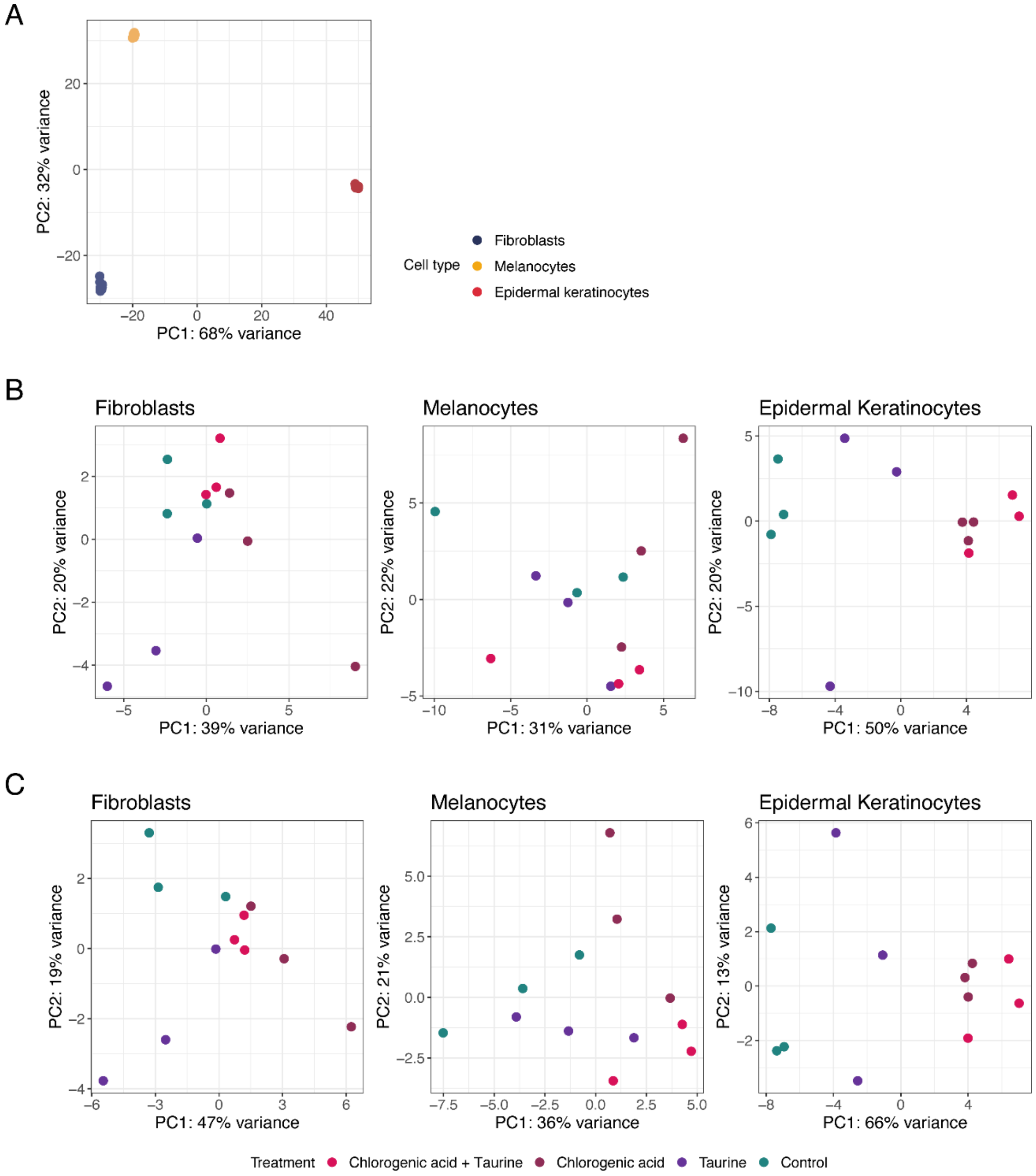

Supplemental Fig. S1. Principal component analysis (PCA) of gene expression. The first two principal components (PCs) of variance-stabilized gene expression are presented. The variances explained by the first PC (PC1) and second PC (PC2) are presented at the x-axis and y-axis, respectively. **A**, PCA of gene expression before strict quality control. Each dot represents a sample from a colored cell type. **B**, PCA of gene expression prior to excluding within-group DEGs. **C**, PCA of gene expression after strict quality control in each cell type. Each dot represents a sample from the colored treatment group.
Abbreviations: DEG, differentially expressed gene.

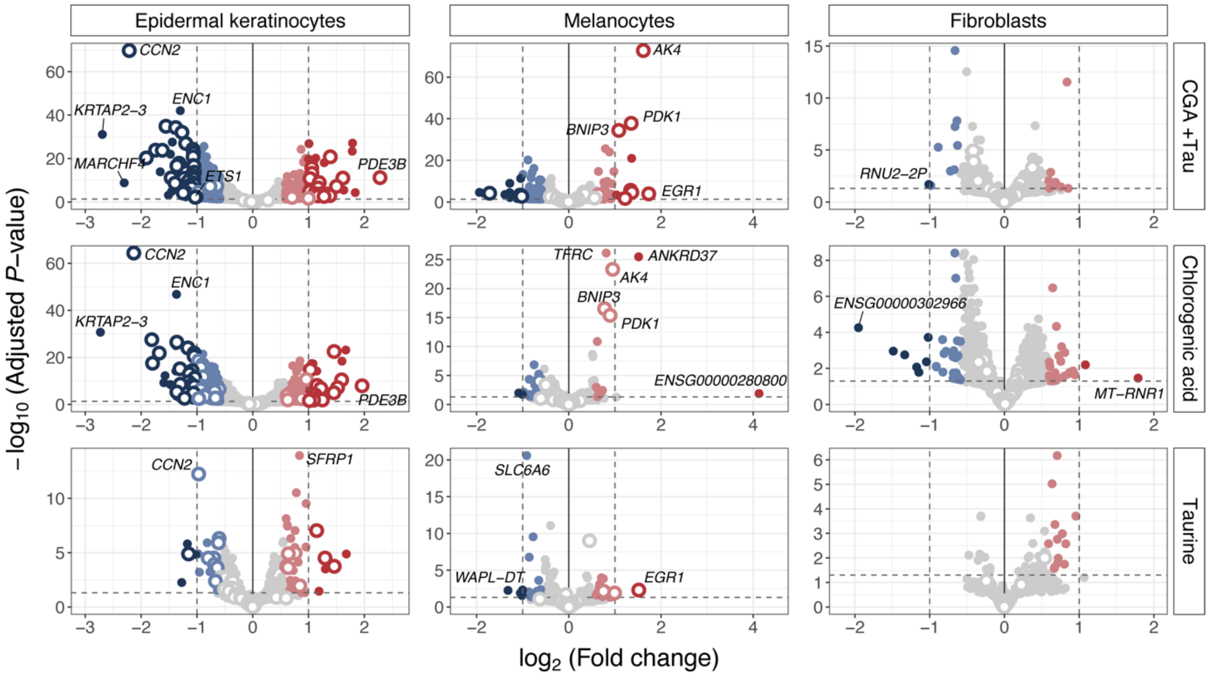

Supplemental Fig. S2. Volcano plots of differential gene expression for each treatment in each human skin cell type. Each dot represents a gene plotted by $\log_2$FC (*x*-axis) and −log10 (adjusted *P*-value) (*y*-axis). Significantly upregulated and downregulated genes (adjusted *P*-value < 0.05 and $|\log_2\text{FC}| > 1$) are highlighted in red and blue, respectively. Genes with suggestive fold changes ($|\log_2\text{FC}| > 0.585$) are displayed in a lighter shade. Selected genes with large expression changes in each cell type are labeled.

Abbreviations: $\log_2$FC, Bayesian shrinkage estimator for $\log_2$ fold change; CGA+Tau, chlorogenic acid + taurine.

A

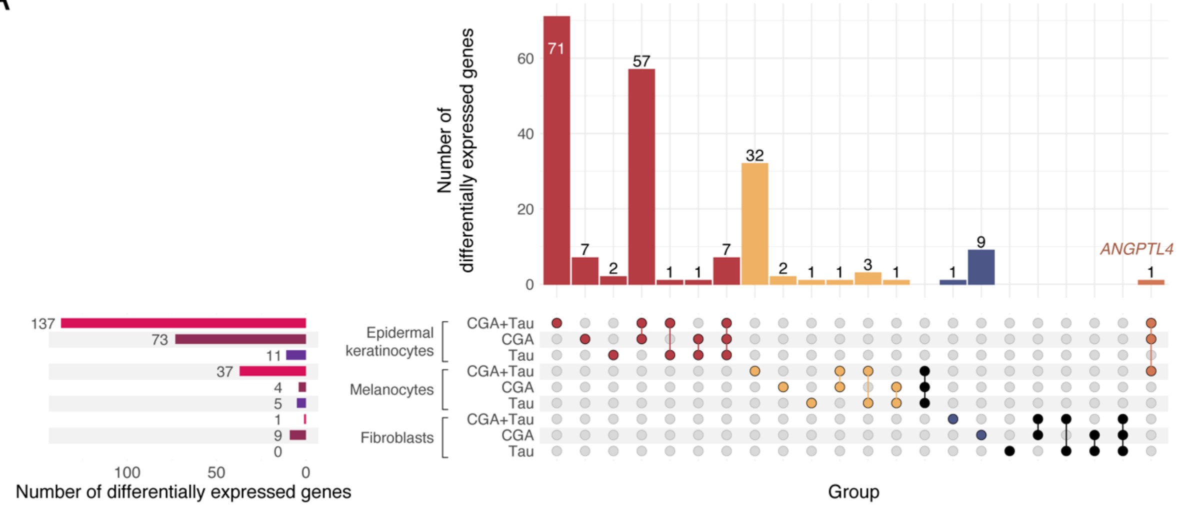

B

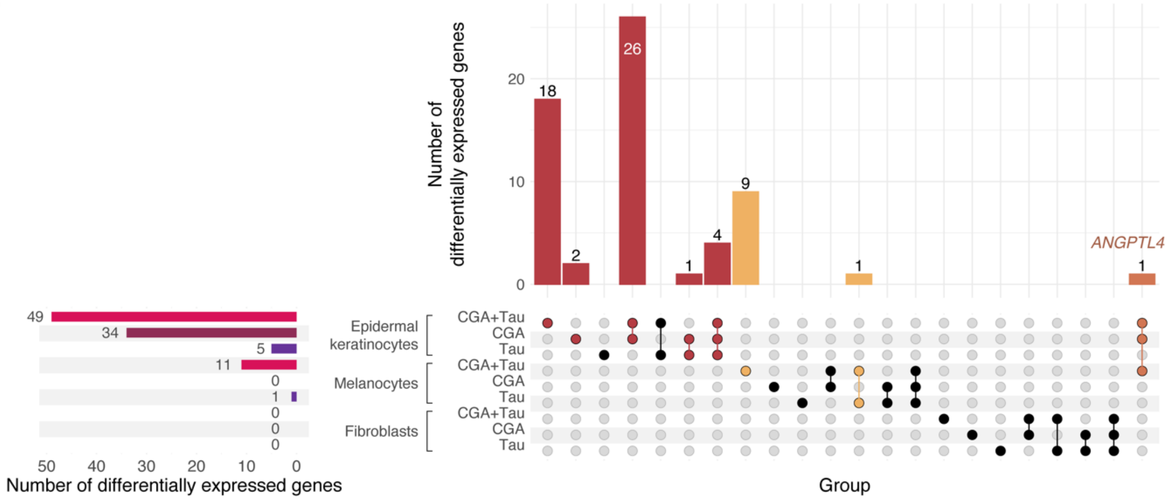

Supplemental Fig. S3. Number of differentially expressed genes (DEGs) identified in this study. **A**, Each bar represents the number of DEGs identified in this study. **B**, Each bar represents the number of aging-related DEGs in this study. Filled dots below the bars indicate the combinations of the conditions contributing to each intersection. The left bar plot shows the total number of DEGs identified in each cell type–treatment condition. ANGPTL4 was the only DEG that responded to the treatment in more than one cell type.
Abbreviations: DEG, differentially expressed gene.

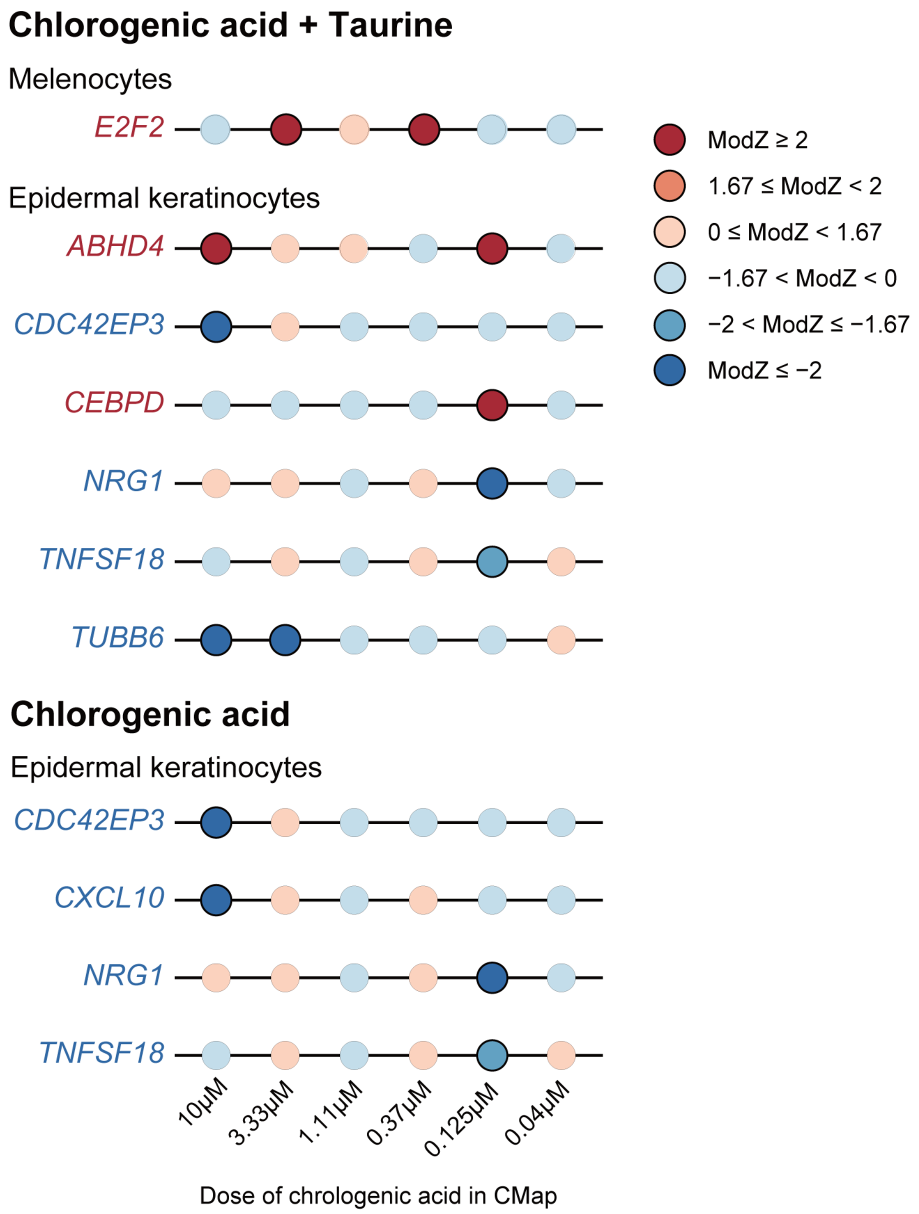

Supplemental Fig. S4. DEGs having supporting evidence from the Connectivity Map (CMap). The genes in red and blue represent DEGs with positive and negative $\log_2$ fold change in this study, respectively. Red and blue dots indicate ModZ value from the CMap for chlorogenic acid treatment across six concentrations (x-axis). Dots with $|ModZ| \geq 1.67$ are outlined in black. Abbreviations: DEG, differentially expressed gene; ModZ, moderated z-score.

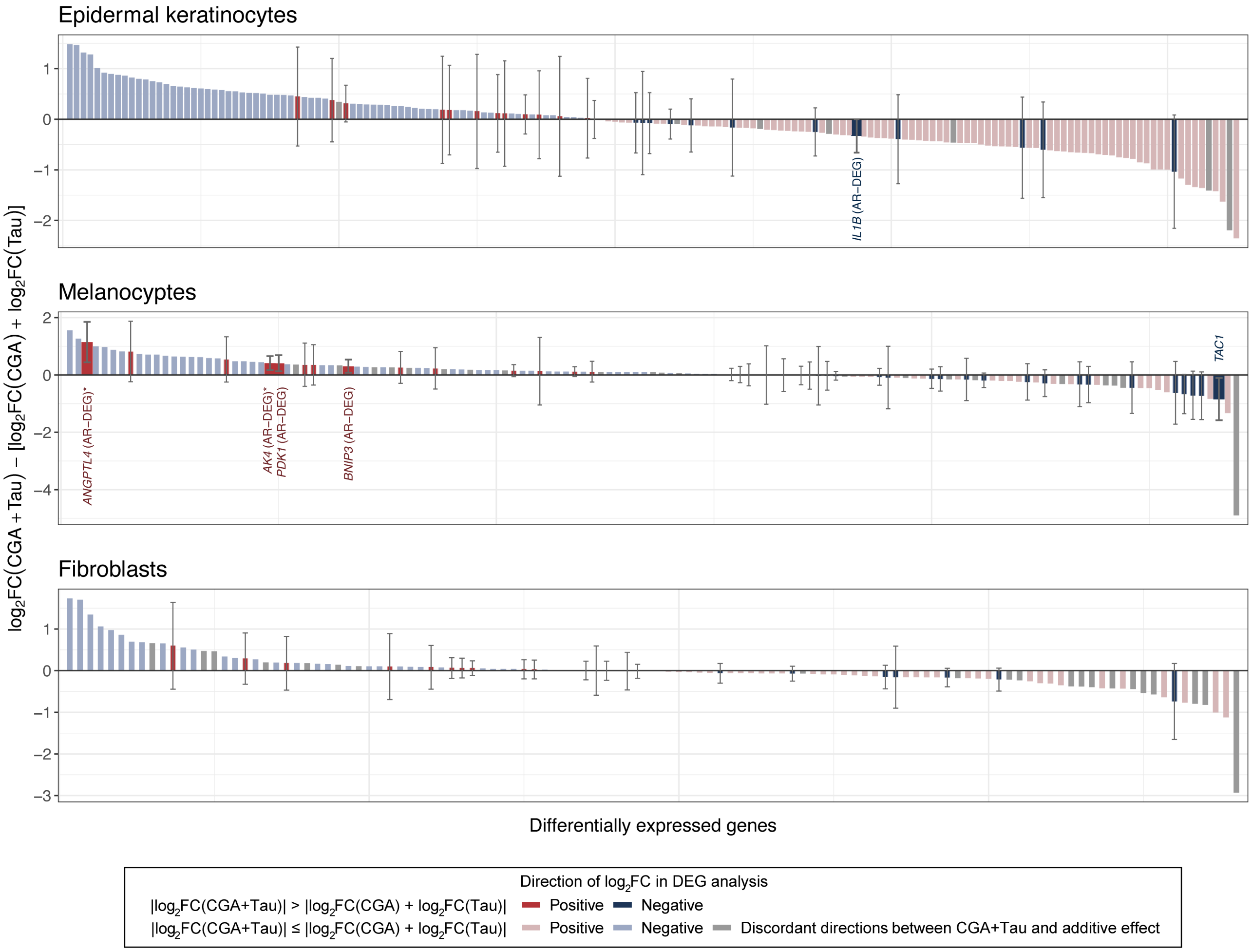

Supplemental Fig. S5. Synergistic effects across the identified DEGs. Each bar represents a DEG plotted by difference between effects of combined treatment and additive effects, defined as $|\log_2FC(CGA+Tau)| - (|\log_2FC(CGA) + \log_2FC(Tau)|$ (using unshrunk $\log_2$ fold change)), on the *y*-axis. Genes on the *x*-axis are ordered by the y-axis value. Genes showing potential synergistic effects ($|\log_2FC(CGA+Tau)| > |\log_2FC(CGA) + \log_2FC(Tau)|$ with consistent effect directions) are highlighted with darker colors and accompanied by 95% confidence intervals. Genes reaching nominal significance level for synergistic effects (*P*-value < 0.05) are indicated by thicker bars and labeled with gene symbols. Genes that passed suggestive significance level after Benjamini–Hochberg multiple testing correction (adjusted *P*-value < 0.1) are marked with an asterisk next to the gene name. AR-DEGs are denoted by "AR-DEG" in parentheses following the gene name.

Abbreviations: DEG, differentially expressed gene; $\log_2FC$, unshrunk $\log_2$ fold change; AR-DEG, aging-related differentially expressed gene; CGA, chlorogenic acid; Tau, taurine; CGA+Tau, combined treatment with chlorogenic acid and taurine.

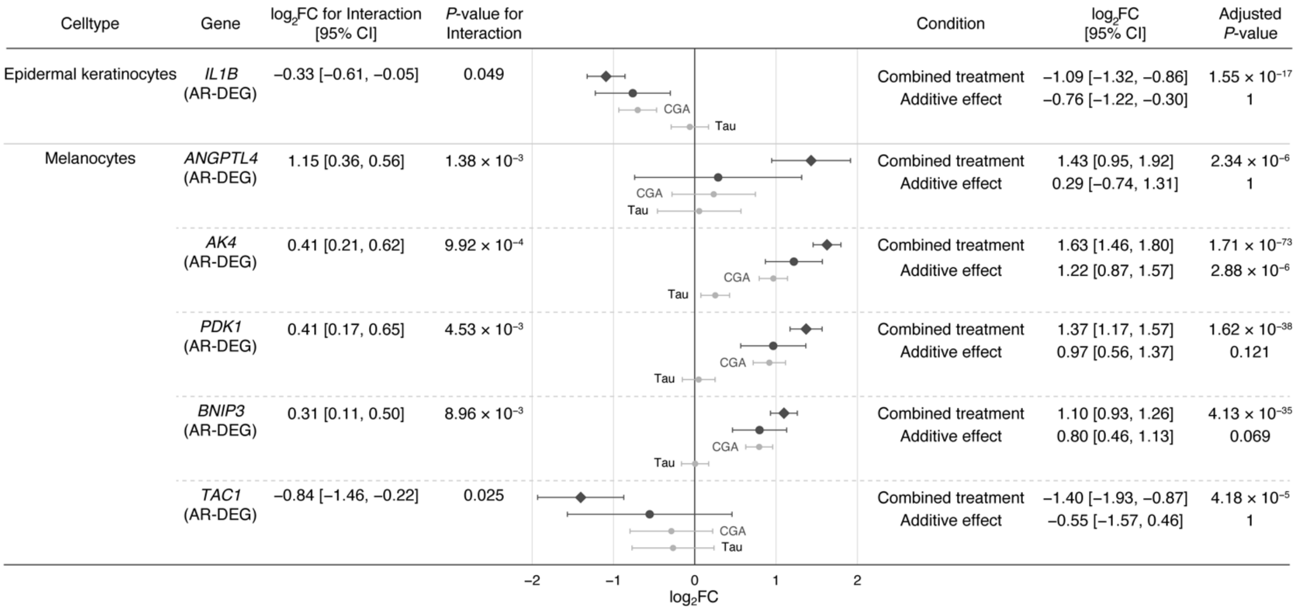

Supplemental Fig. S6. DEGs exhibiting potential synergistic effects. For each gene, $\log_2$ fold changes ($\log_2$FCs) are shown with 95% confidence intervals: the combined treatment effect (black rhombus), the additive effect of chlorogenic acid (CGA) and taurine (black dot), and the individual effects of CGA and taurine (gray dots). The left side of the forest plot displays the $\log_2$FC of the interaction term and the right side displays the $\log_2$FC of the combined treatment effect alongside the additive effect. See Supplemental **Tables S3** and **S4** for detailed summary statistics. Adjusted *P*-values were derived from the Bayesian shrinkage estimator of $\log_2$FC and calculated using Benjamini–Hochberg multiple testing correction based on the total number of genes tested in each cell type (14,111 and 13,986 genes in epidermal keratinocytes and melanocytes, respectively).

Abbreviations: DEG, differentially expressed gene; AR-DEG, aging-related differentially expressed gene; $\log_2$FC, unshrunk $\log_2$ fold change; CI, confidence interval; CGA, chlorogenic acid; Tau, taurine; CGA+Tau, combined treatment with chlorogenic acid and taurine.

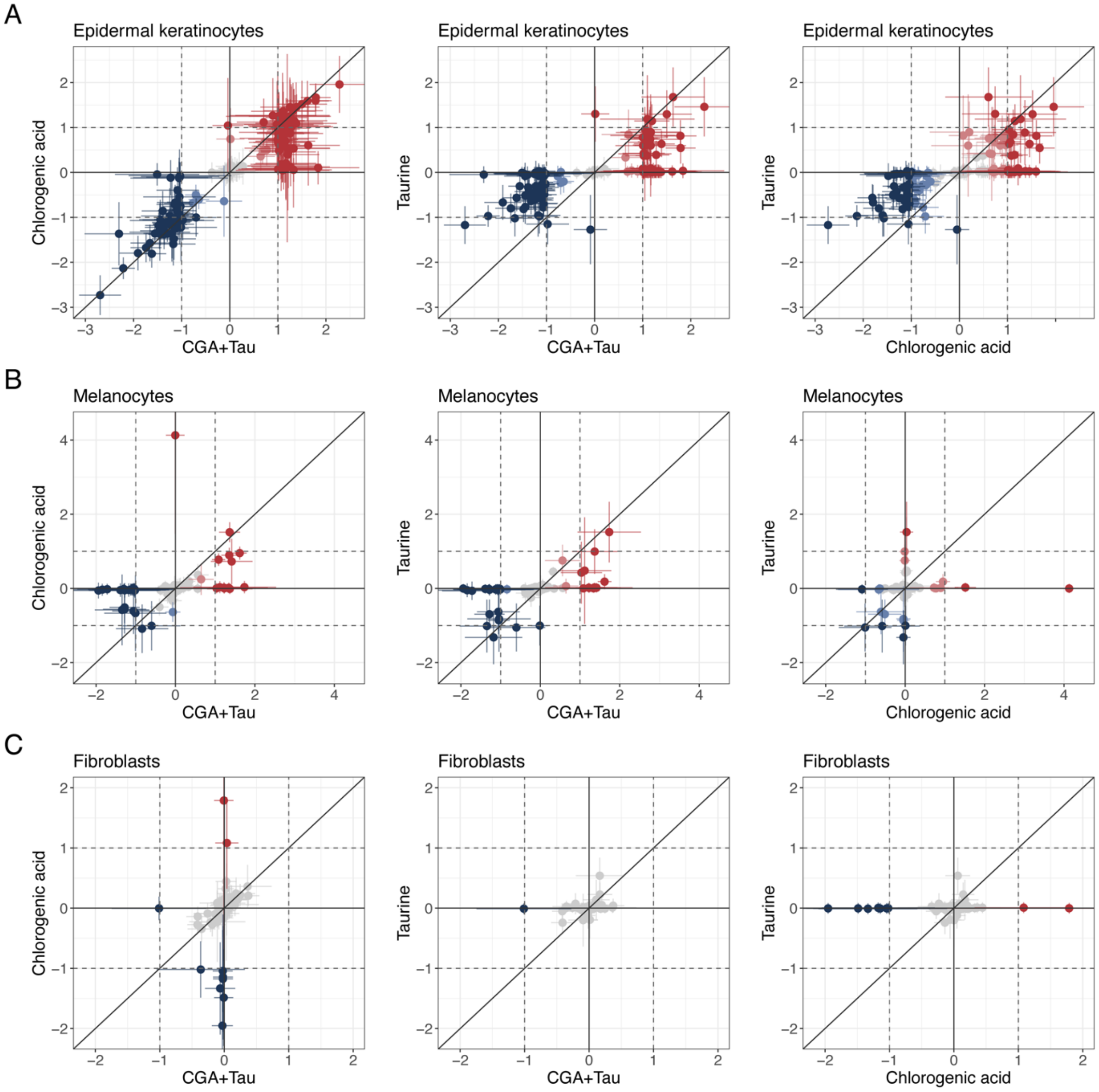

Supplemental Fig. S7. Comparison of $\log_2$ fold changes in the identified DEGs between treatments within each cell type. Genes that were significantly upregulated (red) and downregulated (blue) (adjusted *P*-value < 0.05 and $|\log_2 FC| > 1$) at least one treatment condition are highlighted. Horizontal and vertical lines of each dot indicate the 95% confidence intervals of $\log_2 FC$.

Abbreviations: $\log_2 FC$, Bayesian shrinkage estimator for $\log_2$ fold change.

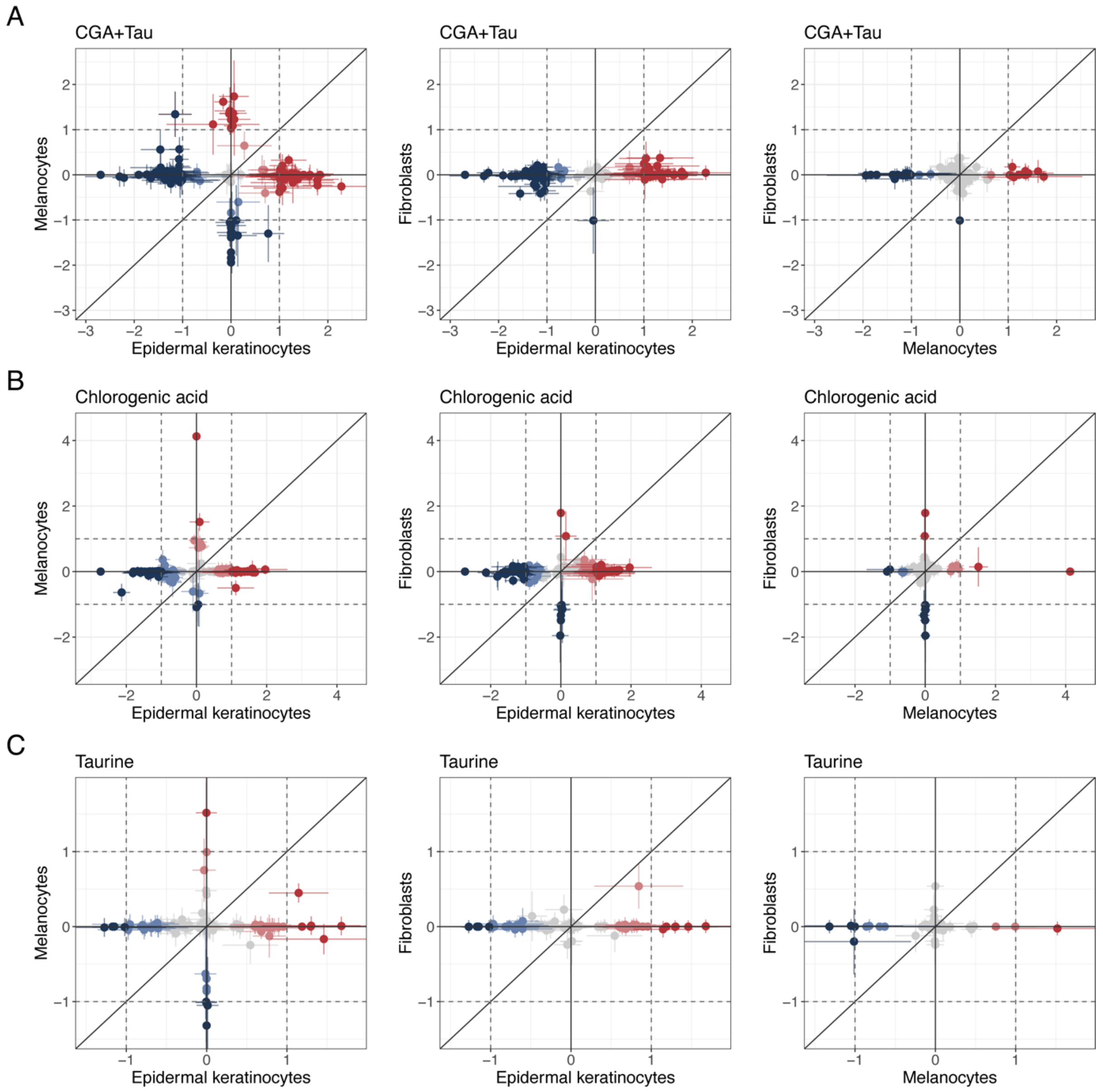

Supplemental Fig. S8. Comparison of $\log_2$ fold changes in the identified DEGs between cell types within each treatment. Genes that were significantly upregulated (red) and downregulated (blue) (adjusted *P*-value < 0.05 and $|\log_2FC| > 1$) in at least one cell type are highlighted. The horizontal and vertical lines of each dot indicate the 95% confidence intervals of $\log_2FC$.

Abbreviations: $\log_2FC$, Bayesian shrinkage estimator for $\log_2$ fold change.

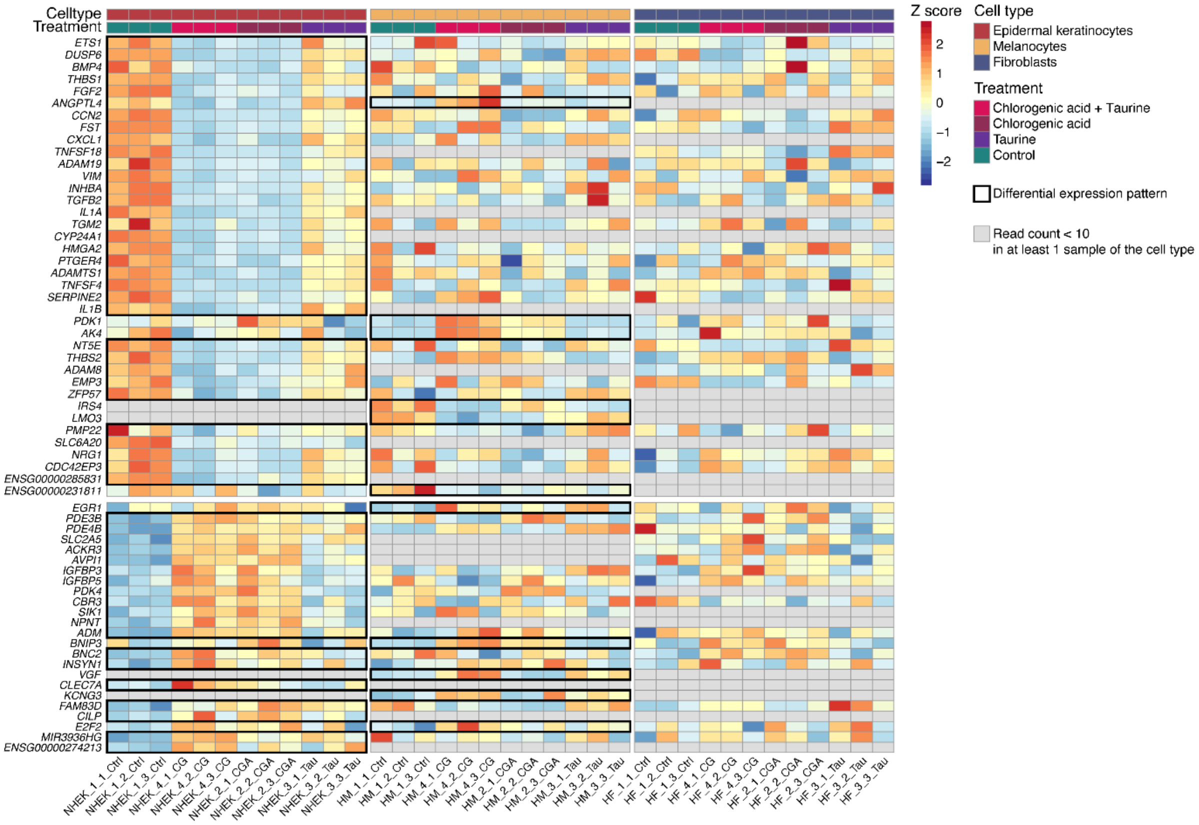

Supplemental Fig. S9. Heatmap showing the expression levels of the 62 aging-related DEGs (AR DEGs). The expression levels were standardized across samples for each cell type. Red and blue indicate higher and lower expression relative to the mean, respectively. Differential expression patterns within each cell type are highlighted by solid black boxes.

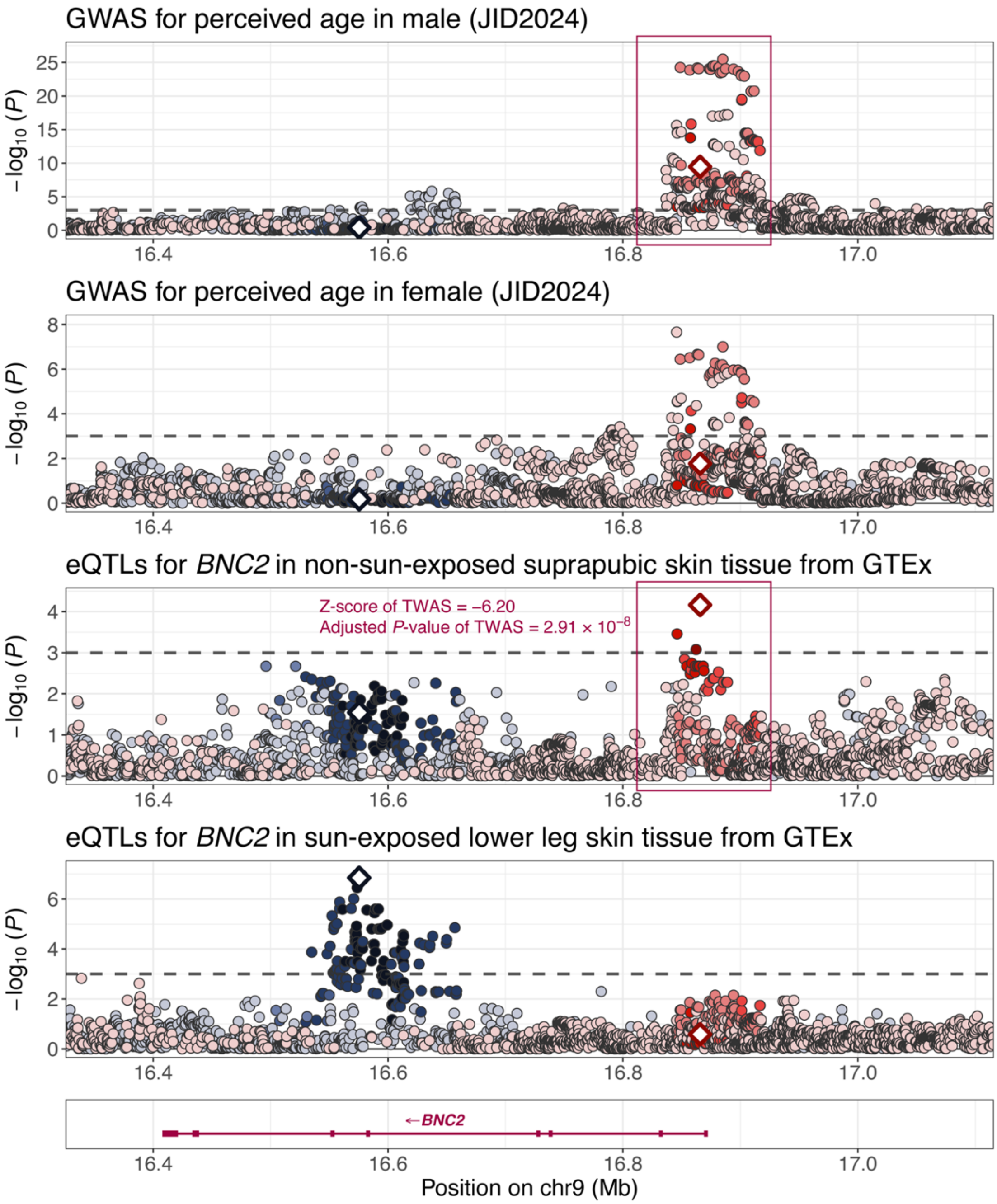

Supplemental Fig. S10. Regional plots of the GWASs for perceived age and eQTLs for *BNC2*. Each dot represents a variant plotted as −log10 (*P*-value) on y-axis against the corresponding variant position (Mb) on the x-axis and is colored according to linkage disequilibrium with the lead variant (rhombus). The Z-score and adjusted *P*-value from the TWAS using FUSION are presented on the plot.

Abbreviations: GWAS, genome-wide association study; eQTL, expression quantitative trait locus; Mb, mega-base pair; chr, chromosome; TWAS, transcriptome-wide association study; GTEx, Genotype-Tissue Expression (GTEx) Project.

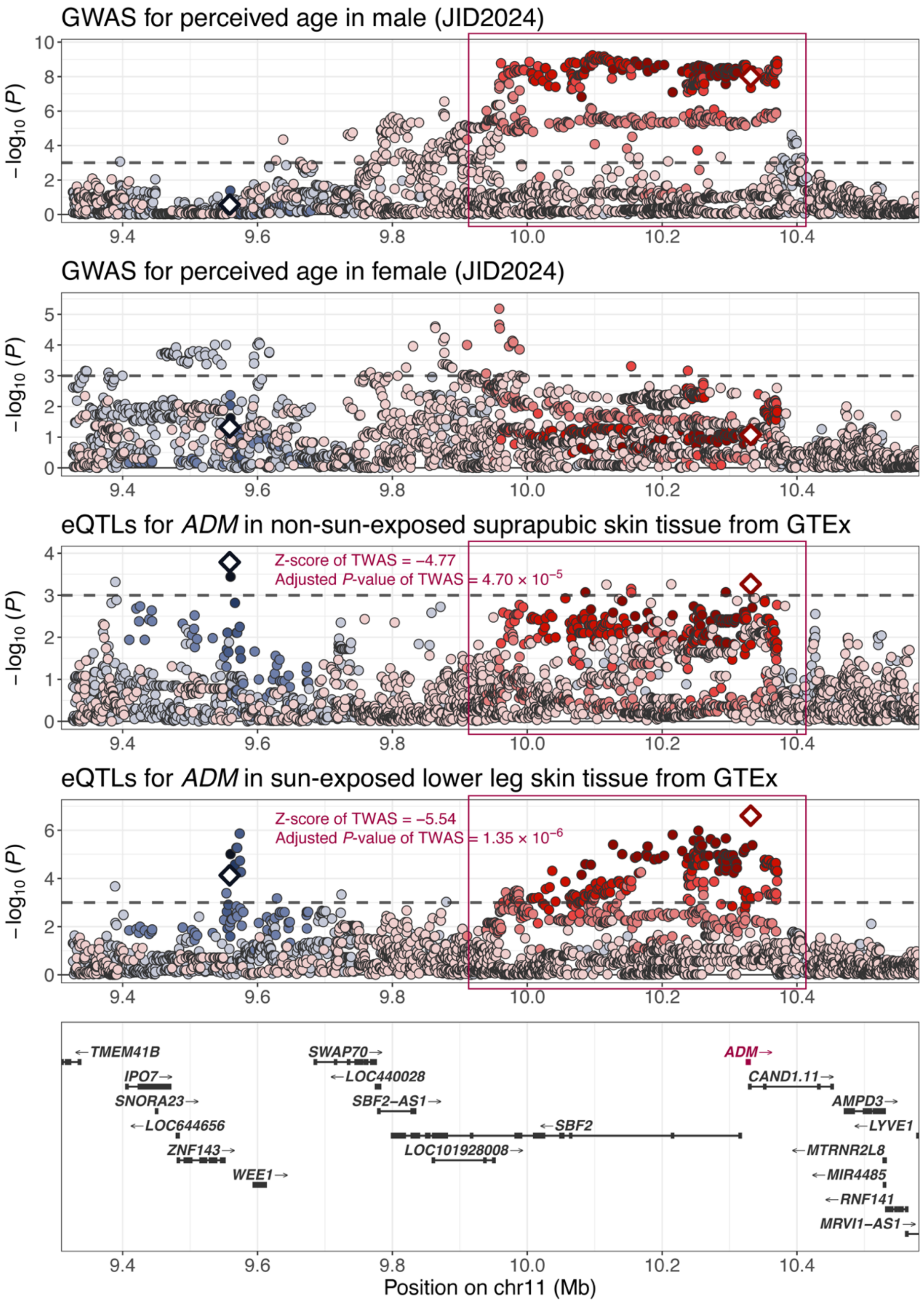

Supplemental Fig. S11. Regional plots of the GWASs for perceived age and eQTLs for *ADM*. Each dot represents a variant plotted as −log10 (*P*-value) on y-axis against the corresponding variant position (Mb) on the x-axis and is colored according to linkage disequilibrium with the lead variant

(rhombus). The Z-score and adjusted *P*-value from the TWAS using FUSION are presented on the plot.
Abbreviations: GWAS, genome-wide association study; eQTL, expression quantitative trait locus; Mb, mega-base pair; chr, chromosome; TWAS, transcriptome-wide association study; GTEx, Genotype-Tissue Expression (GTEx) Project.

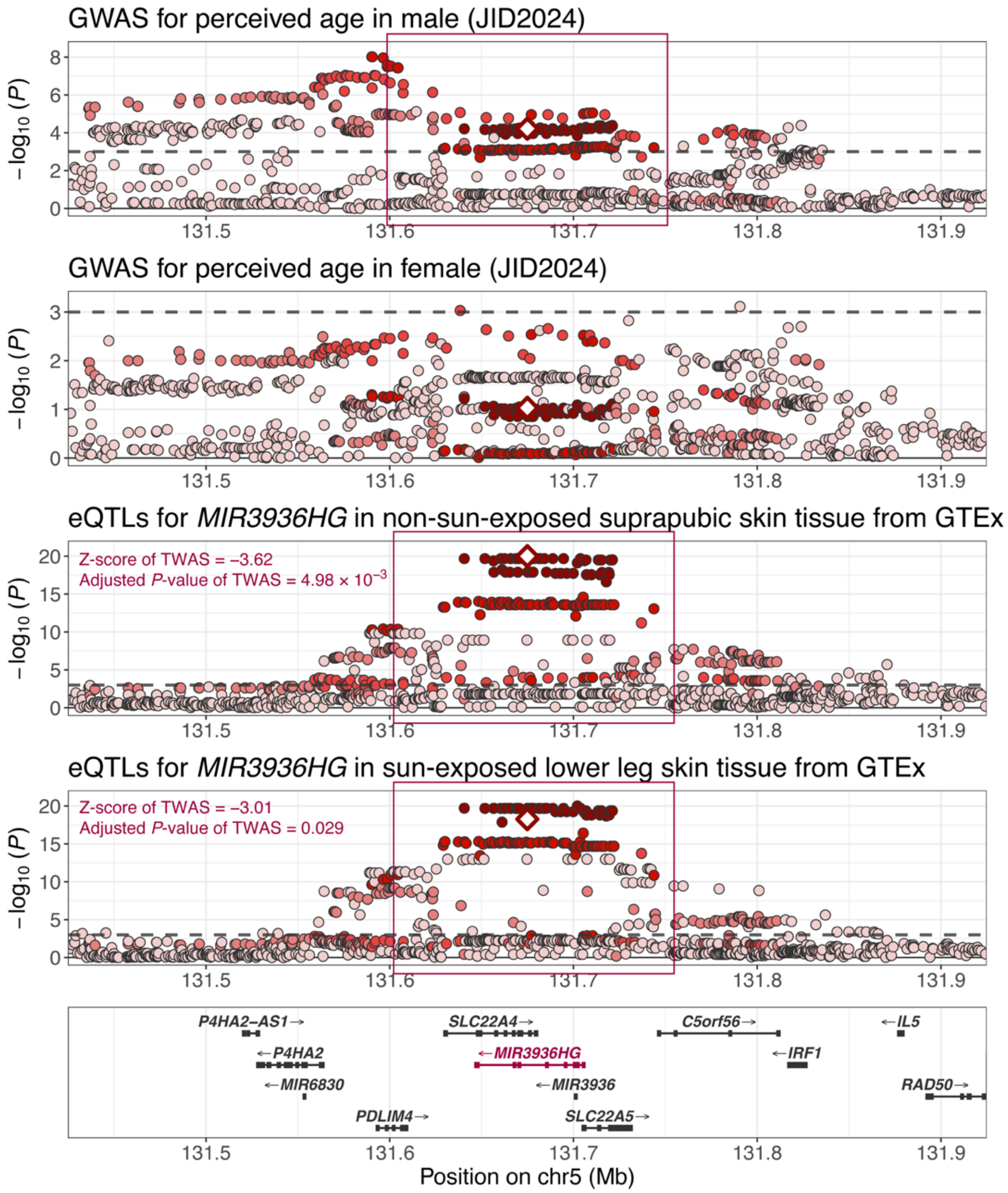

Supplemental Fig. S12. Regional plots of the GWASs for perceived age and eQTLs for *MIR3936HG*. Each dot represents a variant plotted as −log10 (*P*-value) on y-axis against the corresponding variant position (Mb) on the x-axis and is colored according to linkage disequilibrium with the lead variant (rhombus). The Z-score and adjusted *P*-value from the TWAS using FUSION are presented on the plot.

Abbreviations: GWAS, genome-wide association study; eQTL, expression quantitative trait locus; Mb, mega-base pair; chr, chromosome; TWAS, transcriptome-wide association study; GTEx, Genotype-Tissue Expression (GTEx) Project.

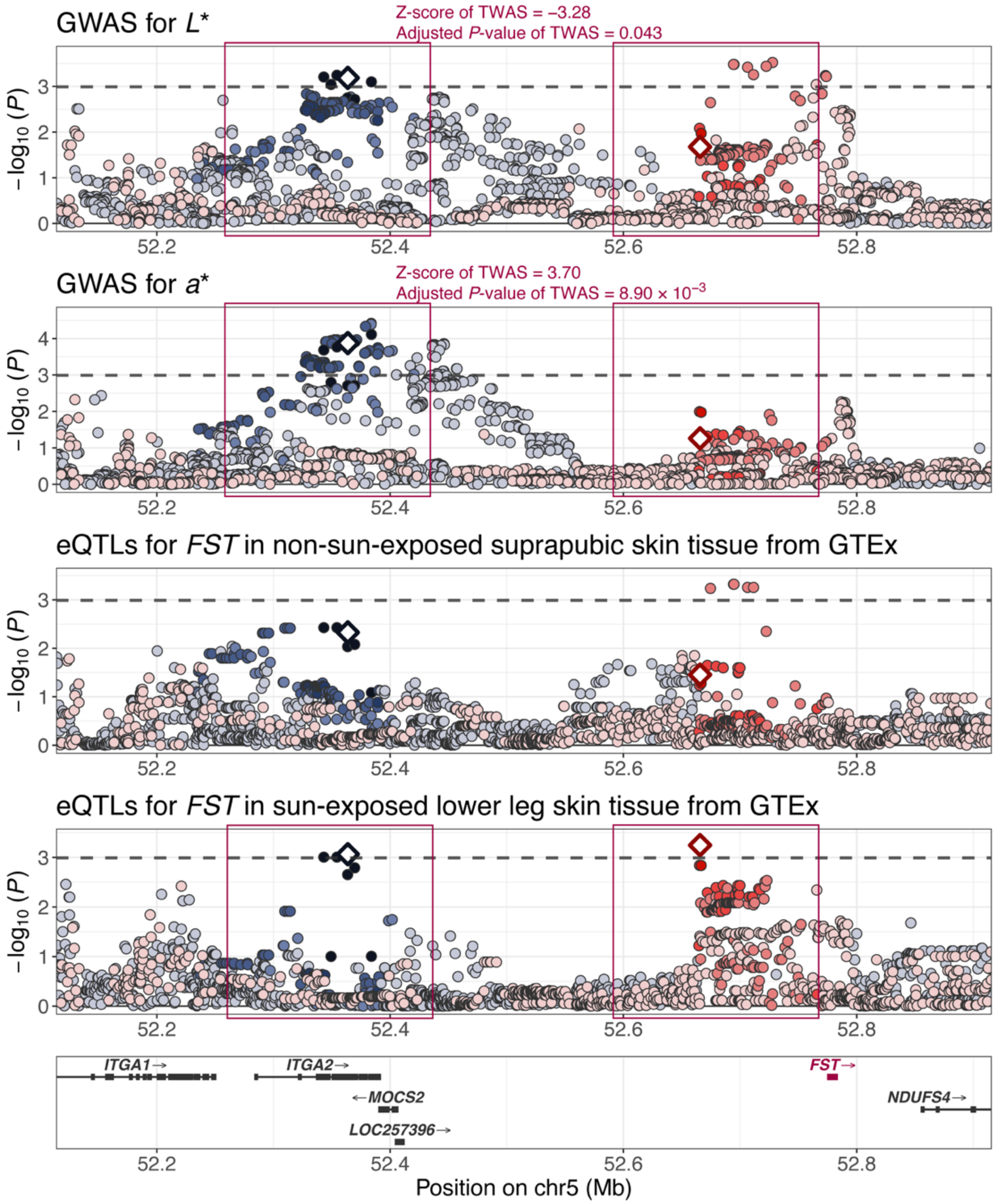

Supplemental Fig. S13. Regional plots of the GWASs for skin color and eQTLs for FST. Each dot represents a variant plotted as −log10 (*P*-value) on y-axis against the corresponding variant position (Mb) on the x-axis and is colored according to linkage disequilibrium with the lead variant (rhombus). The Z-score and adjusted *P*-value from the TWAS using FUSION are presented on the plot.

Abbreviations: GWAS, genome-wide association study; eQTL, expression quantitative trait locus; *L**, CIE LAB value of brightness; *a**, CIE LAB value of redness; Mb, mega base pair; chr, chromosome; TWAS, transcriptome-wide association study; GTEx, Genotype-Tissue Expression (GTEx) Project.

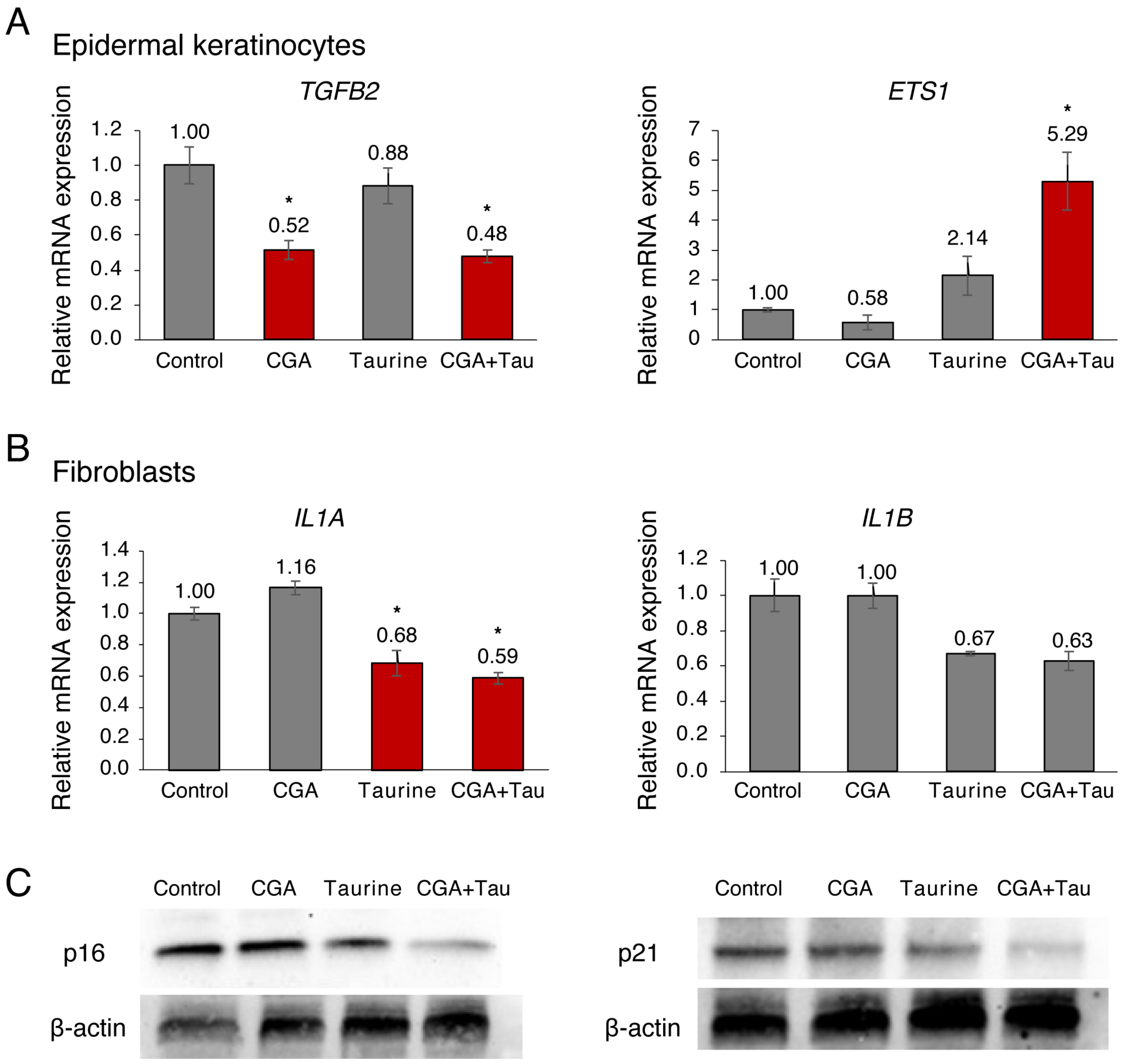

Supplemental Fig. S14. Anti-senescence effects of CGA and taurine and validation of representative AR-DEGs. **A**, Quantitative real time-PCR (RT-PCR) analysis in epidermal keratinocytes. **B**, Quantitative real RT-PCR analysis in fibroblasts. Each bar in panels **A** and **B** represents relative mRNA expression levels compared with the control, shown with 95% confidence interval. Red bars indicate statistically significant differences (* P-value < 0.05). **C**, Western blot analysis of p16 and p21 protein expression in fibroblasts following treatment with CGA, taurine, and their combination (CGA+Tau).

Abbreviations: CGA, chlorogenic acid; CGA+Tau, combined treatment with chlorogenic acid and taurine.
